# Epidemiological, clinical, and virological characteristics of 465 hospitalized cases of coronavirus disease 2019 (COVID‐19) from Zhejiang province in China

**DOI:** 10.1111/irv.12758

**Published:** 2020-05-19

**Authors:** Jiangshan Lian, Xi Jin, Shaorui Hao, Hongyu Jia, Huan Cai, Xiaoli Zhang, Jianhua Hu, Lin Zheng, Xiaoyan Wang, Shanyan Zhang, Chanyuan Ye, Ciliang Jin, Guodong Yu, Jueqing Gu, Yingfeng Lu, Xiaopeng Yu, Dairong Xiang, Lanjuan Li, Tingbo Liang, Jifang Sheng, Yida Yang

**Affiliations:** ^1^ Department of Infectious Diseases State Key Laboratory for Diagnosis and Treatment of Infectious Diseases National Clinical Research Center for Infectious Diseases Collaborative Innovation Center for Diagnosis and Treatment of Infectious Diseases The First Affiliated Hospital College of Medicine Zhejiang University Hangzhou China; ^2^ Department of Gastroenterology the First Affiliated Hospital College of Medicine Zhejiang University Hangzhou China; ^3^ Department of Hepatobiliary and Pancreatic Surgery Key Laboratory of Combined Multi‐Organ Transplantation Division of Hepatobiliary and Pancreatic Surgery the First Affiliated Hospital School of Medicine Zhejiang University Hangzhou China

**Keywords:** coronavirus disease 2019 (COVID‐19), risk factors, severe acute respiratory syndrome coronavirus 2 (SARS‐CoV‐2), Zhejiang province

## Abstract

**Background:**

The severe acute respiratory syndrome coronavirus 2 (SARS‐CoV‐2) and the associated coronavirus disease (COVID‐19) have spread throughout China. Previous studies predominantly focused on its place of origin, Wuhan, causing over estimation of the disease severity due to selection bias. We analyzed 465 confirmed cases in Zhejiang province to determine the epidemiological, clinical, and virological characteristics of COVID‐19.

**Methods:**

Epidemiological, demographic, clinical, laboratory, and management data from qRT‐PCR confirmed COVID‐19 patients from January 17, 2020, to January 31, 2020, were collected, followed by multivariate logistic regression analysis for independent predictors of severe/critical‐type COVID‐19 and bioinformatic analysis for features of SARS‐CoV‐2 from Zhejiang province.

**Results:**

Among 465 COVID‐19 patients, median age was 45 years, while hypertension, diabetes, and chronic liver disease were the most common comorbidities. History of exposure to the epidemic area was present in 170 (36.56%) and 185 (39.78%) patients were clustered in 77 families. Severe/critical‐type of COVID‐19 developed in 49 (10.54%) patients. Fever and cough were the most common symptoms, while diarrhea/vomiting was reported in 58 (12.47%) patients. Multivariate analysis revealed eight risk factors for severe/critical COVID‐19. Glucocorticoids and antibiotics were administered to 60 (12.90%) and 218(46.88%) patients, respectively. Bioinformatics showed four single amino acid mutations and one amino acid position loss in SARS‐CoV‐2 from Zhejiang province, with more similarity to humans than to viruses.

**Conclusions:**

SARS‐CoV‐2 showed virological mutations and more human transmission in Zhejiang province, indicating considerable epidemiological and clinical changes. Caution in glucocorticoid and antibiotics use is advisable.

## 1 INTRODUCTION

An increasing number of cases of severe acute respiratory syndrome coronavirus 2 (SARS‐CoV‐2) pneumonia, known as coronavirus disease (COVID‐19), have set off a global alert and put a heavy public health burden on China due to the rapid transmission ability (basic reproductive number = 2.2) and approximately 11% fatality.^1^, ^2^ When looking back at its occurrence and dissemination, the original serial cases of pneumonia of unknown etiology were reported from Wuhan, China, on December 8, 2019.^3^ On January 7, 2020, a novel coronavirus was identified by the Chinese Center for Disease Control and Prevention (CDC) from the throat swab of a patient and was subsequently named SARS‐CoV‐2 by the WHO.^4^ Though the Chinese government responded rapidly and took drastic measures to stop SARS‐CoV‐2 dissemination, including quarantining Wuhan city on January 23, it spread inevitably, reaching every province of China and beyond to other countries.

Coronaviruses are named for the crown‐like spikes on their surface. People worldwide commonly get infected with the human coronaviruses 229E, NL63, OC43, and HKU1. Two other strains, severe acute respiratory syndrome coronavirus (SARS‐CoV‐2) and Middle East respiratory syndrome coronavirus (MERS‐CoV), are zoonotic in origin and have been linked to lethal diseases.^5^, ^6^ SARS‐CoV‐2 is the seventh newly identified coronavirus with the capacity to infect humans by the Chinese authorities. Though the full sequence, homology to other known coronaviruses, and potential invasion mechanism have been gradually revealed,^7^ there are still many unresolved questions regarding SARS‐CoV‐2. Most importantly, the mutations, transmission ability, virulence changes, and associated clinical features of SARS‐CoV‐2 during dissemination remain unknown.

The first case of COVID‐19 in Zhejiang Province was diagnosed on January 17. Zhejiang province is 1000 kilometers from Wuhan. In contrast to the initial cases from Wuhan who had a history of close contact with Huanan seafood market, the majority of patients in Zhejiang province only had a history of working, studying, or travel to Wuhan. Nevertheless, knowledge about the specific epidemiology and clinical characteristics on a large scale still remains incomplete. In this study, we performed a comprehensive investigation of the epidemiology, clinical, and virological characteristics of 465 patients with laboratory‐confirmed COVID‐19 in Zhejiang province. To the best of our knowledge, this is by far the largest number of COVID‐19 patients reported in Zhejiang province with distinct clinical features compared to Wuhan.

## 2 METHODS

### 2.1 Data sources and ethics

We performed a retrospective study focusing on the epidemiological, clinical, and virological characteristics of confirmed cases of COVID‐19 from January 17, 2020, to January 31, 2020. The data were uniformly collected by the Health Commission of Zhejiang Province. All patients were assigned to specific hospitals for unified treatment according to Zhejiang Province's emergency rule. All patients who were diagnosed with COVID‐19 based on the WHO interim guidance were successfully enrolled in this study.^4^ All data pertaining to the included cases have been shared with WHO, and the primary analytic results were immediately reported to the authorities of Zhejiang province. Since case collection and analysis were determined by the Health Commission of Zhejiang province under national authorization and considered to be a part of the continuing public health outbreak investigation, our study was regarded as exempt from institutional review board approval.

For in‐depth data analysis, the subtype definition of COVID‐19 patients was according to the Chinese diagnosis and treatment scheme for SARS‐CoV‐2 (5th edition) with minor modification based on the WHO standards. Specifically, the severity of COVID‐19 was categorized as mild, common, severe, or critical. Mild type was defined as mild symptoms with no pneumonia on imaging. Common type was defined as having respiratory tract symptoms and pneumonia on imaging. Severe type was characterized by dyspnea, respiratory rate ≥30/minute, blood oxygen saturation ≤93%, PaO2/FiO2 ratio <300, and/or lung infiltrates >50% within 24‐48 hours. Critical cases were those that exhibited respiratory failure, septic shock, and/or multiple organ dysfunction/failure.

### 2.2 Procedures

We obtained epidemiological, demographic, clinical, laboratory, management, and outcome data from the patients’ medical records. Clinical outcomes were followed up to January 31, 2020. If data were missing from the records or clarification was needed, we obtained data by direct communication with the attending doctors and other healthcare providers. All data were checked by at least two doctors. Laboratory confirmation of SARS‐CoV‐2 was done in the following institutions: the Zhejiang province CDC, the first affiliated hospital, School of Medicine, Zhejiang University, and the local CDC at the city level. Throat swab specimens from the upper respiratory tract and sputum samples that were obtained from all patients at admission were maintained in a viral‐transport medium. SARS‐CoV‐2 infection was confirmed by real‐time reverse transcription‐polymerase chain reaction (RT‐PCR) using the same protocol described previously.^8^ Other respiratory viruses including influenza A virus (H1N1, H3N2, H7N9), influenza B virus, respiratory syncytial virus, parainfluenza virus, adenovirus, SARS‐CoV, and MERS‐CoV were also examined with routine real‐time RT‐PCR. Possible causative infection with bacteria or fungi in sputum or endotracheal aspirates was also investigated at admission in P3 level laboratories. All patients underwent chest X‐rays or chest computed tomography (CT) at admission.

### 2.3 Outcomes

We collected and described the epidemiological data (ie, living in Wuhan and returning to Hangzhou, traveling back from Wuhan, contact with people from Wuhan and Hubei province, exposure to Wuhan seafood market within 14 days before illness onset); anthropometrics; demographics; symptoms and signs on admission; laboratory and chest X‐ray/CT results; comorbidity; coinfection with other respiratory pathogens; treatment (including drugs, intensive care, and mechanical ventilation); and clinical outcomes.

### 2.4 Viral sequencing, phylogenetic analysis, and synonymous codon usage analysis

All published sample sequences (n = 23) were obtained from the NCBI viral genome database and NGDC (https://bigd.big.ac.cn/ncov/), except for ZJ01 which was the name of the SARS‐CoV‐2 isolated separately from Zhejiang patients. The coronavirus samples came from all over the world, including Wuhan (n = 4), Guangdong (n = 3), USA (n = 2), and Australia (n = 1). There were also SARS specimens (n = 3) and other animal coronaviruses (n = 10). Multalin (http://multalin.toulouse.inra.fr/multalin/multalin. html) was used to compare the differences between these sequences. Further SWISS‐MODEL online server (https://swissmodel.expasy.org/) was used to reconstruct the three‐dimensional structure of protein according to gene or amino acid sequence. The reconstructed protein model was put in PyMol to explore molecular mechanic and protein function. Phylogenetic tree establishment, evolutionary analysis, and other bioinformatic analyses were performed by MEGA7.0. The heat map of relative synonymous codon usage (RSCU) was drawn with MeV 4.9.0, where Codon W1.4.2 was used to estimate the RSCU of these coronaviruses. Hierarchical clustering was used to analyze RSCU of these strains, with utilization of Euclidean distance selection and average linkage clustering.

### 2.5 Statistical analysis

For continuous variables, mean (SD) and median (IQR) were used for normally and abnormally distributed data. Categorical variables were expressed as numbers (%). For laboratory results, it was determined whether the measurement was outside the normal range. Univariate logistic regression analysis was used to identify the risk factors of severe/critical‐type patients. All significant variables in univariate analysis were included in a multivariate logistic regression model with Forward:Wald method to identify independent predictors of severe/critical‐type patients. No adjustment for multiple testing was performed. A two‐sided *P* value of less than 0.05 was considered statistically significant, and SPSS (version 26.0) was used for all analyses.

## 3 RESULTS

### 3.1 Demographic and epidemiologic characteristics

This study analyzed 465 patients with confirmed SARS‐CoV‐2‐infected pneumonia from January 17, 2020, to January 31, 2020, in Zhejiang province. As shown in Table 1, the median age of the patients was 45 years (range, 5 to 88 years); the majority of the infections were in patients aged 15‐49 years. There was no significant difference in the number of male and female patients. Among them, 224 (48.17%) patients were self‐employed and 60 (12.90%) were current smokers. A total of 29.68% of patients had at least one coexisting medical condition, with top three being hypertension, diabetes, and chronic liver disease. Based on the available data from 322 patients who had a definite history of exposure to the epidemic area (Wuhan), we found that 170 (52.8%) patients had a confirmed history of contact with patients from the epidemic area. After taking into account the detailed exposure time and date of illness onset, we calculated that the median incubation period was four days (range, 1 to 13).

**Table 1:** Demographic and epidemiologic characteristics of 465 patients with SARS‐CoV‐2 infection in Zhejiang, China

Characteristic	Value
Age	
Median (range)‐y	45(5‐88)
0‐14 y	3 (0.65%)
15‐49 y	293 (63.01%)
50‐64 y	138 (29.68%)
≥65 yr	31 (6.67%)
Sex	
Female	222 (47.74%)
Male	243 (52.26%)
Occupation	
Agricultural worker	103 (22.15%)
Self‐employed	224 (48.17%)
Employee	94 (20.22%)
Retired	31 (6.67%)
Other	13 (2.80%)
Current smoker	60 (12.90%)
Coexisting condition	
Any	138 (29.68%)
Hypertension	82 (17.63%)
Diabetes	28 (6.02%)
Chronic liver disease	19 (4.09%)
Cancer	5 (1.08%)
Chronic renal disease	5 (1.08%)
Heart disease	3 (0.65)
Pregnancy	2 (0.43%)
COPD	0
Immunosuppression	0
Exposure History	
From Wuhan	322 (69.25%)
Contact with patients	170 (36.56%)
Cluster	185 (77 families)
Incubation period median‐range(days)	4 (1‐13)
Clinical type on admission	
Mild type (no pneumonia)	20 (4.30%)
Common type	396 (85.16%)
Severe type	41 (8.82%)
Critical type	8 (1.72%)

Abbreviation: COPD, chronic obstructive pulmonary disease.

The situation in Zhejiang province presented the scenario of family clustering, where we found 185 infected patients from 77 families. For rare case analysis, two pregnant women were diagnosed with COVID‐19 in our study. Both of them were mild type, with one in the first trimester and the other in the last trimester. One 5‐year‐old child was diagnosed with mild‐type COVID‐19 due to family exposure. In contrast to SARS, MERS, and H7N9, most COVID‐19 cases were diagnosed as common type according to the SARS‐CoV‐2 Chinese (5th edition) guidelines. Only 8.82% patients developed the severe type, and 1.72% patients progressed to the critical type, showing lower tendency severity when compared to data from Wuhan.

### 3.2 Clinical Features and laboratory abnormalities

The clinical characteristics of the patients are shown in Table 2. Briefly, fever and cough were the most common symptoms. A total of 85.81% patients had fever with median maximum temperature at admission of 38°C, while 53.88% had temperature below 38°C, supporting caution for patients with moderate fever. Additionally, 67.10% patients had a cough, but the sputum production rate was only 33.55%. Therefore, dry cough was a typical symptom. Diarrhea or vomiting was reported in 12.47% of the patients, higher than the results from Wuhan. On admission, 149 (32.04%) patients had leukocytopenia, 78 (16.77%) had lymphocytopenia, five (1.08%) had thrombocytopenia, 69 (14.84%) had increased international normalized ratio (INR), and 170 (36.56%) had decreased albumin level. The elevated levels of ALT and AST were observed in 47 (10.11%) and 52 (11.18%) patients, respectively. Creatine kinase (CK) was increased in 23 (4.95%) patients, and lactate dehydrogenase (LDH) was increased in 117 (25.16%) patients.

**Table 2:** Clinical characteristics and selected laboratory abnormalities observed in 465 patients infected with SARS‐CoV‐2

Characteristic		Value
Fever		
	Any‐no. (%)	399 (85.81%)
	Subgroup‐no. (%)	
	≤38.0	215 (53.88%)
	38.1‐39.0	148 (37.09%)
	>39.0	36 (9.02%)
Cough		312 (67.10%)
Sputum production		156 (33.55%)
Hemoptysis		9 (1.94)
Sore throat		75 (16.13%)
Nasal obstruction		27 (5.81%)
Muscle ache		51 (10.97%)
Fatigue		95 (20.43%)
Shortness of breath		22 (4.73%)
Diarrhea		36 (7.74%)
Nausea and vomiting		22 (4.73%)
Headache		50 (10.75%)
Blood routine		Patients (n = 465)
Leukocytes (×10^9^/L; normal range 4‐10)	Mean (SD)	4.97 (1.78)
	Increased	7 (1.51%)
	Decreased	149 (32.04%)
Neutrophils (×10^9^/L; normal range 2‐7)	Mean (SD)	3.40 (3.66)
	Increased	15 (3.23%)
	Decreased	94 (20.22%)
Lymphocytes (×10^9^/L; normal range 0.8‐4)	Mean (SD)	1.24 (0.51)
	Increased	0 (0%)
	Decreased	78 (16.77%)
Platelets (×10^9^/L; normal range 83‐303)	Mean (SD)	184 (62.38)
	Decreased	5 (1.08%)
Hemoglobin (g/L; normal range: men 131‐172, women 113‐151)	Mean (SD)	138.83 (15.81)
	Decreased	38 (8.17%)
Hematocrit (%; normal range: men 38‐50.8, women 33.5‐45)	Mean (SD)	40.88 (4.47)
	Decreased	75 (16.13%)
Coagulation function		
International normalized ratio (INR, normal range 0.85‐1.15)	Mean (SD)	1.05 (0.11)
	Increased	69 (14.84%)
Blood biochemistry		
Albumin (g/L; normal range 40‐55)	Mean (SD)	41.21(4.44)
	Decreased	170 (36.56%)
Alanine aminotransferase (U/L; normal range 9‐50)	Mean (SD)	27.95 (22.49)
	Increased	47 (10.11%)
Aspartate aminotransferase (U/L; normal range 15‐40)	Mean (SD)	28.37(17.56)
	Increased	52 (11.18%)
Total bilirubin (µmol/L; normal range 0‐26)	Mean (SD)	11.52 (7.02)
	Increased	13 (27.96%)
Serum sodium (mmol/L; normal range 137‐147)	Mean (SD)	138.35 (3.65)
	Increased	2 (0.43%)
	Decreased	125 (26.88%)
Serum potassium (mmol/L; normal range 3.5‐5.3)	Mean (SD)	3.82 (0.41)
	Increased	1 (0.22%)
	Decreased	89 (19.14%)
Blood urea nitrogen (mmol/L; normal range 3.1‐8)	Mean (SD)	3.97 (1.60)
	Increased	8 (1.72%)
	Decreased	135 (29.03%)
Serum creatinine (µmol/L; normal range: men 57‐97, women 41‐73)	Mean (SD)	70.43 (25.91)
	Increased	57 (12.26%)
	Decreased	28 (6.02%)
Creatine kinase (U/L; normal range 50‐310)	Mean (SD)	112.93 (180.20)
	Increased	23 (4.95%)
Lactate dehydrogenase (U/L; normal range 120‐250)	Mean (SD)	231.36 (185.08)
	Increased	117 (25.16%)
Glucose (mmol/L; normal range 3.9‐6.1)	Mean (SD)	6.22 (2.13)
	Increased	168 (36.13%)
	Decreased	5 (1.08%)
Infection‐related biomarkers		
Procalcitonin (ng/mL; normal range 0‐0.5)	Mean (SD)	0.06 (0.05)
	Increased	0 (0%)
Erythrocyte sedimentation rate (mm/h; normal range: men 0‐15, women 0‐20)	Mean (SD)	22.69 (19.87) (From 202 patients)
	Increased	114 (56.44%)
C‐reactive protein (mg/L; normal range 0‐8)	Mean (SD)	15.98 (2.16)
	Increased	234 (50.32%)
Chest x‐ray/ CT findings		N = 462
	Normal	54 (11.69%)
	Unilateral pneumonia	99 (21.43%)
	Bilateral pneumonia	177 (38.31%)
	Multiple mottling and ground‐glass opacities	132 (28.57%)

Abbreviations: CT, computed tomography; SD, standard deviation.

**Figure 1:**
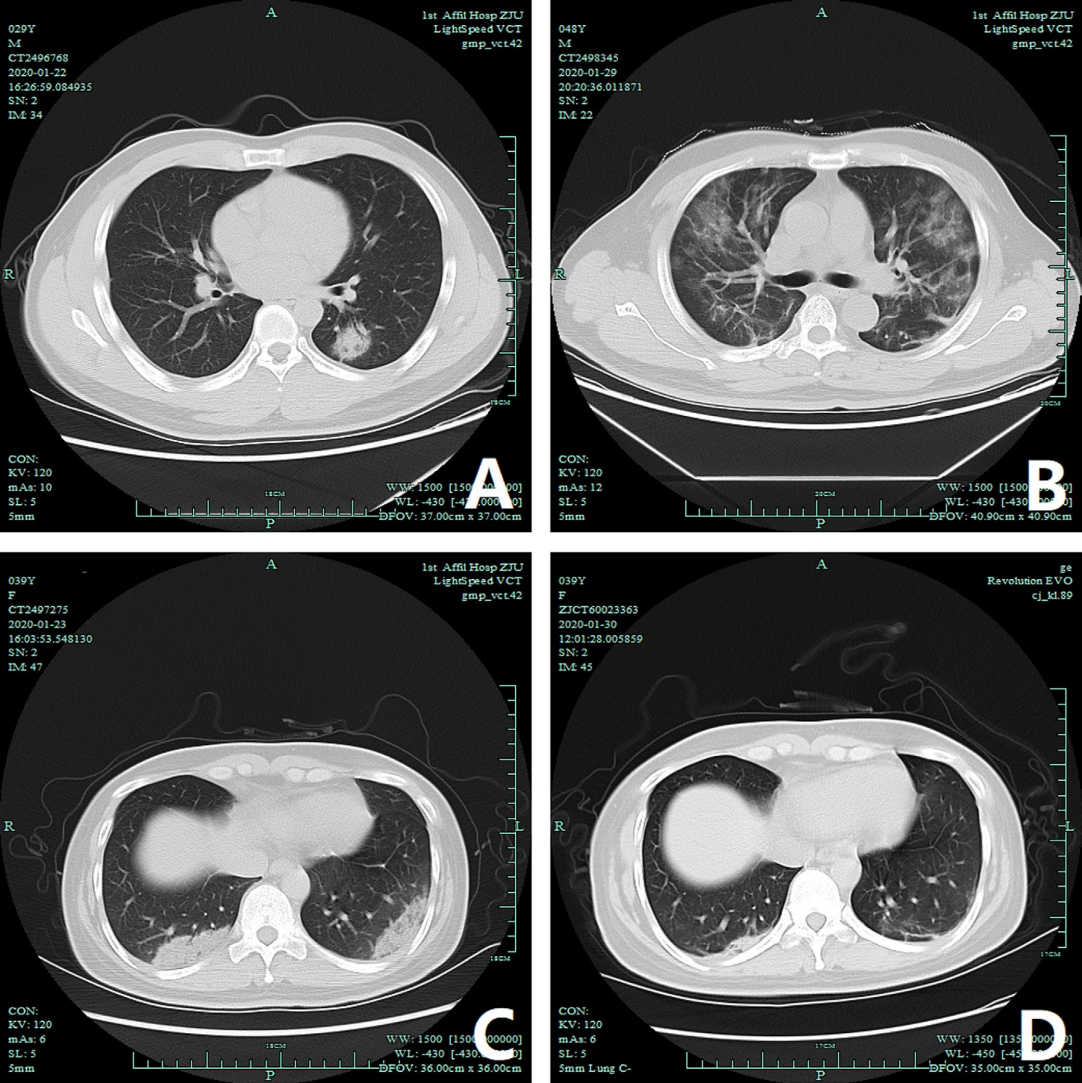
Chest computed tomography (CT) images of three patients diagnosed with COVID‐19. (A) Non‐contrast axial CT of a 30‐year‐old man diagnosed with COVID‐19 pneumonia (mild type), in the lung window, demonstrates a solid nodule in the left lower lobe. The bronchus is visible as an air bronchogram. (B) Non‐contrast axial CT of a 48‐year‐old man diagnosed with COVID‐19 pneumonia (severe type) and ARDS, in the lung window, demonstrates peripheral and multiple ground‐glass opacities (GGO). The pulmonary architecture, including vasculature and bronchi, can be still seen. (C‐D) Non‐contrast axial CT of a 40‐year‐old man diagnosed with COVID‐19 pneumonia (severe type), in the lung window, demonstrates bilateral subpleural consolidation and opacities in the two lower lobes, more densely consolidated on the right. CT lung of the same patient one week after treatment, in the lung window demonstrates that the lung lesions were significantly absorbed

Concerning infection‐related parameters, over 50% patients had increased erythrocyte sedimentation rate (ESR) and C‐reactive protein (CRP). Intriguingly, none of the patients had increased procalcitonin (PCT) levels. Since radiography is pivotal for disease identification and diagnosis, 462 patients underwent chest radiography on admission, among which 408 (88.31%) were diagnosed with pneumonia. Bilateral ground‐glass opacities and consolidation were the most common radiological findings inEdits our cases (Figure 1).

### 3.3 Treatment and complications

All patients were treated in isolation with supportive and empiric medication. As show in Table 3, a total of 387 (83.23%) patients received antiviral treatment, including interferon‐α sprays, arbidol hydrochloride capsules (2 tablets three times daily), or lopinavir and ritonavir 2 tablets (500 mg) twice daily orally. Triple therapy was administered to 142(36.70%) patients consisting of interferon‐α sprays plus lopinavir/ritonavir plus arbidol. Furthermore, 115 (29.72%) patients received interferon‐α sprays + lopinavir/ritonavir double therapy, while 32 (8.27%) patients received lopinavir/ritonavir + arbidol double therapy. Additionally, 39 (10.08%) received lopinavir/ritonavir single therapy. Overall, 57.63% patients received antiviral treatment within four days after illness onset.

**Table 3:** Complications and treatment in 465 patients with SARS‐CoV‐2 infection

Variable	Value
	No. of patients (%)
Complications	
Acute respiratory distress syndrome	11(2.37%)
Shock	1 (0.22%)
Liver injury	61(13.12%)
Treatment	
Anticoronavirus treatment	387 (83.23%)
Interferon‐α + lopinavir/ritonavir + arbidol	142 (36.70%)
Interferon‐α + lopinavir/ritonavir	115 (29.72%)
Lopinavir/ritonavir	39 (10.08%)
Lopinavir/ritonavir + arbidol	32 (8.27%)
Time from onset of illness to administration of antiviral therapy	
0‐2 days	115 (29.72%)
3‐4 days	108 (27.91%)
5‐6 days	70 (18.09%)
>6 days	94 (24.29%)
Oxygen therapy	445 (95.70%)
Mechanical ventilation	
Non‐invasive	4 (0.86%)
Invasive	4 (0.86%)
CRRT	0
ECMO	0
Glucocorticoids	60 (12.90%)
Time from onset of illness to Glucocorticoid administration (days, mean ± SD)	6.93 ± 3.34
Dosage (mg) (interquartile range)	40 (40‐80)
IVIG	42 (9.03%)
Dosage (g) (interquartile range)	20 (16.25‐20)
Antibiotic treatment	218 (46.88%)
Admission to intensive care unit	4（0.86%）

Abbreviations: CRRT, continuous renal replacement therapy; ECMO: extracorporeal membrane oxygenation; IVIG: intravenous immunoglobulin.

We had our own experience of using glucocorticoids and antibiotics for treatment. In contrast to the previous Wuhan reports, we had lower glucocorticoid administration rates, and 60 (12.90%) patients received methylprednisolone within 6.93 ± 3.34 days of onset of illness with daily dosage of 40 to 80 mg. Besides, only 218 (46.88%) patients received antibiotics therapy, which is much lower than the 71% from the Wuhan 99 cases study.^1^ The antibiotics used included cephalosporins, quinolones, carbapenem, tigecycline against methicillin‐resistant *Staphylococcus aureus*, and linezolid. Antifungal drugs were used when appropriate.

Oxygen therapy plays an important role in the supportive care of patients. In this study, 445 (95.70%) patients received oxygen therapy, and eight patients underwent mechanical ventilation (four non‐invasive and four invasive). Only five patients were admitted to intensive care unit (ICU), where four patients received invasive mechanical ventilation till January 31 for 4‐9 days. The ventilator was set at P‐SIMV mode, with inhaled oxygen concentration of 35%‐100% and positive end‐expiratory pressure of 6‐12 cm H_2_O. None of the patients underwent continuous blood purification due to renal failure or extracorporeal membrane oxygenation (ECMO). Liver injury was the most common complication (61 patients), followed by acute respiratory distress syndrome (ARDS) (11 patients) and shock (one patient). By the end of the study period on January 31, all patients had survived and were being treated in hospital, except for one patient who was discharged.

### 3.4 Risk factor prediction for severe/critical COVID‐19

There were total 49 patients with severe/critical COVID‐19 in this study. When compared with mild and common COVID‐19, initial univariate analysis of epidemiological, clinical, and laboratory variables identified 25 significant risk factors for severe/critical COVID‐19 (Table S1). Based on these variables, further multivariate logistic regression analysis with forward selection method was performed and we found that male sex, any coexisting disease, cough, muscle ache, diarrhea, decreased lymphocytes, increased CRP, and decreased albumin were the independent risk factors for severe/critical COVID‐19 (Table 4).

**Table 4:** Multivariate analysis of risk factors of 49 severe/critical‐type COVID‐109 patients

Risk Factor	Odds Ratio (95% CI)	*P* value
Sex (Female)	0.332 (0.150‐0.739)	.007
Any coexisting disease	3.066 (1.454‐6.465)	.003
Cough	2.600 (1.013‐6.671)	.047
Muscle ache	5.717 (2.284‐14.307)	.000
Diarrhea	4.580 (1.677‐12.511)	.003
Lymphocytes	0.236 (0.087‐0.641)	.005
C‐reactive protein (CRP)	1.016 (1.003‐1.029)	.013
Albumin	0.850 (0.774‐0.933)	.001

Abbreviation: CI, confidence interval.

### 3.5 Multiple sequence alignment and bioinformatics analysis

**Figure 2:**
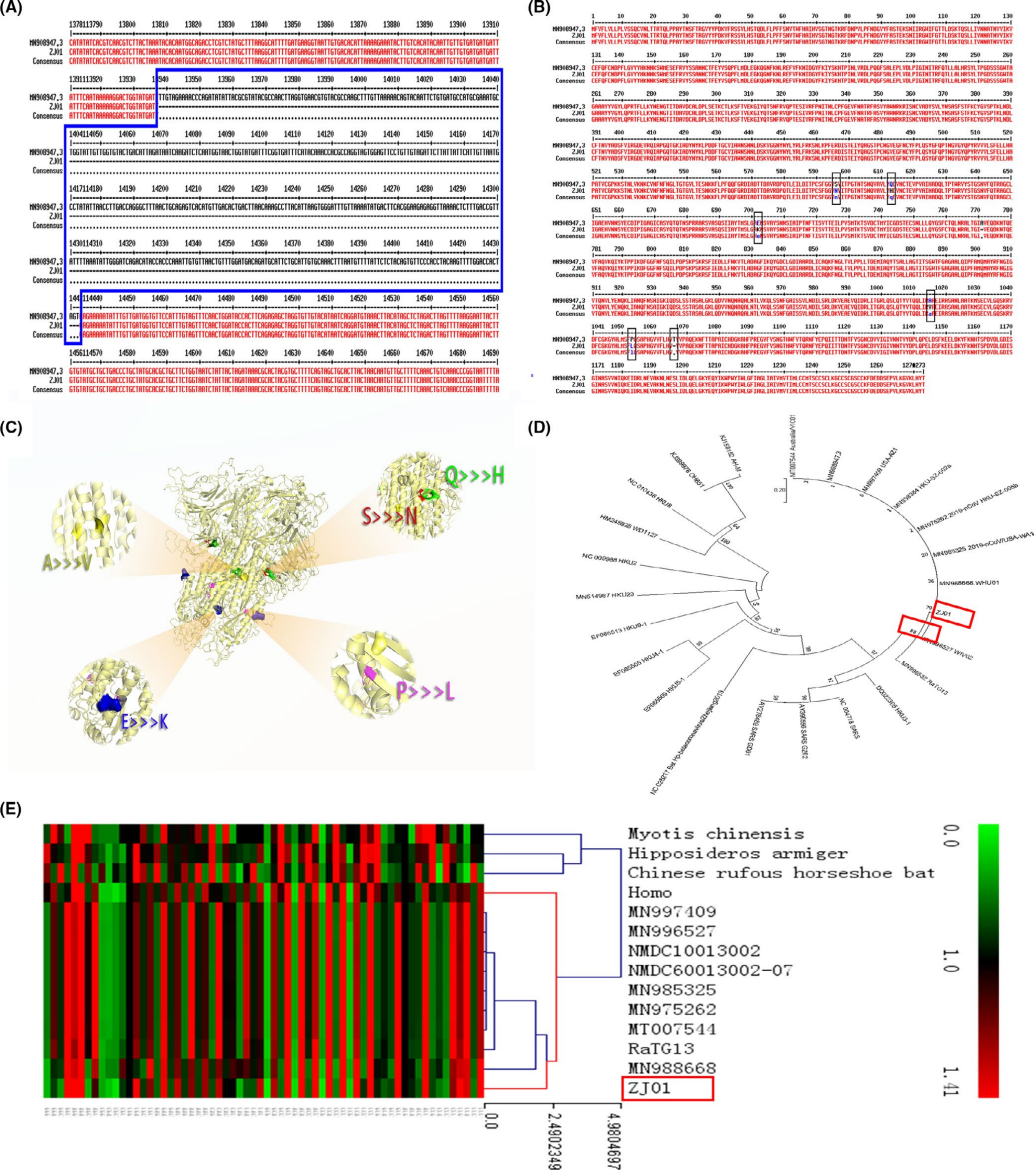
Viral sequencing, phylogenetic analysis, and synonymous codon usage analysis of ZJ01. (A) Due to an accident during sequencing, the ORF1ab segment of the ZJ01 sample lost a 0.5 k base during this study. (B) The amino acid sequence alignment of ZJ01 shows that it is nearly identical with the Wuhan SARS‐CoV‐2 in S protein. (C) A three‐dimensional reconstruction model of the S protein clearly shows where the mutations are located. (D) Maximum likelihood genealogy shows the evolution of SARS‐CoV‐2. It reveals the relationship between SARS‐CoV‐2 and other coronaviruses. (E) Results from the RSCU heat map demonstrate that the RSCU of ZJ01 appears to be moving away from its relatives

We confirmed that ZJ01 is a SARS‐CoV‐2 with 29,381 bases by utilizing biological methods (Appendix S1). However, in the process of sequencing, we failed to perfectly measure its entire genome, resulting in the deletion of a 0.5 KB gene segment encoding ORS 1aboratory (Figure 2A **).** The S protein of SARS‐CoV‐2 is responsible for recognizing angiotensin‐converting enzyme 2 (ACE2) receptor and mediating membrane‐enveloped fusion for invasion. By sequencing the S protein base of ZJ01, we found that ZJ01 was very similar to MN908947.3 from Wuhan **(**Figure 2B **)**. There were only four single amino acid mutations and one amino acid position loss in this sequence, as shown by 3D protein reconstruction (Figure 2C **)**. Except for one mutation point exposed on the protein surface (blue), the other mutations occurred within the S protein conformation.

The evolutionary tree revealed the evolutionary path of the coronavirus family (Figure 2D **)**. Though there were variations among the members of the SARS‐CoV‐2 family, they were not enough to allow them to evolve into additional subspecies. The most surprising result was the RSCU heat map (Figure 2E). RSCU refers to the relative probability that a specific codon encodes a corresponding amino acid in a synonymous codon. ZJ01’s RSCU is more similar to humans than other viruses. This phenomenon is also seen in MN988668 which was found in Wuhan. This suggests that the SARS‐CoV‐2 is beginning to evolve after a period of human‐to‐human transmission.

## 4 DISCUSSION

In this study, we analyzed 465 confirmed cases of COVID‐19 in Zhejiang province to determine the epidemiological, clinical, and virological characteristics of COVID‐19. We found that the patients in Zhejiang province were younger and had less comorbidities than those reported by Zhou and Yu et al from Wuhan.^9^ However, the plentiful data from Wuhan may cause selection and publication bias as far more severe patients were enrolled in hospitals due to insufficient health care. Moreover, SARS‐CoV‐2 itself may undergo a virulence change during dissemination, inducing alterations in epidemiological and clinical features of COVID‐19 patients. Therefore, it is necessary and pivotal to perform an extended descriptive study on the epidemiological, clinical, and virological characteristics of the SARS‐CoV‐2 infection in other areas of China apart from Wuhan, Hubei province. Except for one report about a familial cluster of six COVID‐19 patients in Shenzhen,^10^ our study results also present the largest family clustering data focusing on SARS‐CoV‐2 infection status out of Wuhan. Therefore, we believe that our results are representative of the virus situation in China apart from Wuhan.

The findings in this series of patients suggest that SARS‐CoV‐2 can cause severe illness such as ARDS (2.37%) and shock (0.22%), at a much lower rate than the previously reported rates of 17% of ARDS and 4% of shock.^11^ Furthermore, the ICU admission rate of COVID‐19 was also much lower in Zhejiang province at 0.86%, compared with study in Wuhan that 23 (23%) out of 99 were admitted to ICU.^1^ All these results indicate that the virulence of SARS‐CoV‐2 may decrease during dissemination. We also discovered changes in several complications and symptoms. For instance, the rate of diarrhea, nausea, and vomiting was higher at 12.47%, while liver injury was a newly recognized feature with a rate of 13.12%. Since 20%‐25% of patients with MERS‐CoV or SARS‐CoV‐2 infection are known to have diarrhea,^12^ the change in digestive symptoms observed in patients in Zhejiang province hints at the possibility of a virus mutation leading to an increased affinity for the GI tract.

SARS‐CoV‐2 is an enveloped virion with approximately 50‐200 nm diameter and a single positive‐sense RNA genome.^13^ The envelope spike (s) protein is capable of mediating receptor binding and membrane fusion,^14^ vital for determining host tropism and transmission capacity.^15^, ^16^ Since intestinal epithelial cells have a high ACE2 level^17^ which is the target of SARS‐CoV‐2, it is plausible that the virus could invade the GI tract through this pathway. Another case study has also reported the identification of the SARS‐CoV‐2 nucleotide in patient feces.^18^ Consequently, we compared the sequence of SARS‐CoV‐2 from Zhejiang with that from Wuhan and other regions. When compared to other regions, we found a slight mutation of the S protein of SARS‐CoV‐2 from Zhejiang and more similarity of the RSCU of ZJ01 to humans, suggesting that the SARS‐CoV‐2 may begin to evolve after a period of human‐to‐human transmission. Future research should focus on the influence of S protein change on the virus conformation and biological functions as well as the influence of potential evolution on the virulence and transmission capacity of SARS‐CoV‐2.

The incubation period and family clustering were found to be important for COVID‐19 control and prevention, which has been rarely reported in previous studies. According to latest available data based on 10 confirmed cases, the mean incubation period was estimated to be 5.2 days (95% confidence interval [CI]: 4.1 to 7.0).^2^ In contrast, our results, based on 170 cases, identified a median incubation period of four days (range, 1 to 13). We also found that 185 of 465 (39.78%) patients were clustered in 77 families, reinforcing the previously reported family clustering phenomenon.^10^ Since we could not calculate the basic reproductive number (R0) in this study, we applied a modified Boltzmann sigmoid function to predict the rate of increase and total number of COVID‐19 cases in Zhejiang province.

The risk factors for severe/critical COVID‐19 were calculated by multivariate logistic regression analysis. The eight factors are presented in Table 4. In summary, male sex, any coexisting disease, symptoms of cough, muscle ache, and diarrhea, laboratory abnormalities of decreased lymphocyte/albumin and increased CRP level are risk factors for severe/critical COVID‐19. One feature of SARS‐CoV‐2 reported from Wuhan is its high fatality rate, which caused widespread panic. Therefore, timely and accurate treatment is very important. Till January 31, we had no patient deaths and only 1.72% critical cases out of the enrolled 465 patients. Compared with the Wuhan treatment experience, we had a relatively lower glucocorticoid prescription rate and were more cautious with antibiotics administration, which may be valuable for further investigation in a cohort study.

There are several limitations of this study that need to be acknowledged. Firstly, the retrospective nature of this study may decrease its credibility and future prospective cohort studies should be considered. Secondly, a complete analysis of COVID‐19 on a national level is urgently needed, which might provide more solid data. Thirdly, although we summarized the risk factors for severe/critical type of COVID‐19, there is still lack of a prediction model for disease fatality, since most of the enrolled cases are currently under treatment. Finally, cytokine change is common in coronavirus infections^19^ and has been reported in a previous SARS‐CoV‐2 study.^8^ Therefore, it would be better if we could also test cytokine changes in our study.

In summary, Zhejiang province was the first province that exhibited the highest response level for SARS‐CoV‐2 infection and quarantined every suspected patient, followed by immediate virus detection. All these measures were effective and helped to control virus dissemination. On the basis of our experience with the virus, we revealed the novel epidemiological, clinical, and virological features of SARS‐CoV‐2 as summarized in this paper. The changes in these features may be due to virus mutation during dissemination, as we observed on comparison of the viral sequence between Zhejiang and Wuhan with focus on the invasion‐related S protein. We have also provided large‐scale preliminary data pertaining to our treatment experience, including low and appropriate glucocorticoid usage, early and timely antiviral therapy, avoiding unnecessary antibiotics, and ample oxygen supply. Further in‐depth analysis on the therapeutic interventions is under way.

## AUTHOR CONTRIBUTION

**Jiangshan Lian:** Conceptualization (equal); Data curation (equal); Methodology (equal); Project administration (equal); Writing‐original draft (lead). **Xi Jin:** Data curation (equal); Formal analysis (equal); Methodology (equal); Writing‐original draft (equal). **Shaorui Hao:** Investigation (equal); Methodology (equal); Software (equal); Visualization (equal). **Hongyu Jia:** Data curation (equal); Methodology (equal); Software (equal); Visualization (equal). **Huan Cai:** Formal analysis (equal); Methodology (equal); Software (equal); Visualization (equal). **Xiaoli Zhang:** Formal analysis (equal); Visualization (equal). **Jianhua Hu:** Methodology (equal). **Lin Zheng:** Investigation (equal); Methodology (equal). **Xiaoyan Wang:** Data curation (equal). **Shanyan Zhang:** Software (equal). **Chanyuan Ye:** Data curation (equal); Validation (equal). **Ciliang Jin:** Formal analysis (supporting); Methodology (supporting). **Guodong Yu:** Data curation (supporting); Formal analysis (supporting); Methodology (supporting). **Jueqing Gu:** Data curation (supporting); Investigation (supporting); Visualization (supporting). **Yingfeng Lu:** Formal analysis (supporting); Investigation (supporting). **Xiaopeng Yu:** Validation (supporting); Visualization (supporting). **Dairong Xiang:** Formal analysis (supporting); Validation (supporting). **Lanjuan Li:** Conceptualization (equal); Investigation (equal); Project administration (equal); Resources (equal); Writing‐review & editing (equal). **Tingbo Liang:** Conceptualization (equal); Project administration (equal); Supervision (equal); Writing‐review & editing (equal). **Jifang Sheng:** Conceptualization (equal); Project administration (equal); Resources (equal); Supervision (equal); Writing‐review & editing (equal). **Yida Yang:** Conceptualization (lead); Funding acquisition (lead); Project administration (equal); Resources (lead); Supervision (lead); Writing‐review & editing (lead).

## Supporting information

Supplementary Material
