# Supplementary material for: Epidemiological, clinical, and virological characteristics of 465 hospitalized cases of coronavirus disease 2019 (COVID‐19) from Zhejiang province in China
Source: Influenza Other Respir Viruses. 2020 May 19;14(5):564–74. doi: 10.1111/irv.12758 (PMC7273099; doi:10.1111/irv.12758)
Supplement: Supplementary file 1 — Supplementary Material [file IRV-14-564-s001.docx]

**Supplementary Appendices**

**Epidemiological, clinical, and virological characteristics of 465 hospitalized cases of coronavirus disease 2019 (COVID-19) from Zhejiang province in China**

Jiangshan Lian^1†^, Xi Jin^2†^, Shaorui Hao^1†^, Hongyu Jia^1†^, Huan Cai^1†^, Xiaoli Zhang^1^, Jianhua Hu^1^, Lin Zheng^1^, Xiaoyan Wang^1^, Shanyan Zhang^1^, Chanyuan Ye^1^ Ciliang Jin^1^, Guodong Yu^1^, Jueqing Gu^1^, Yingfeng Lu^1^, Xiaopeng Yu^1^, Dairong Xiang^1^, Lanjuan Li^1^*, and Tingbo Liang^3^* and Jifang Sheng^1^* and Yida Yang^1^*

Jiangshan Lian, Xi Jin, Shaorui Hao, Hongyu Jia and Huan Cai contributed equally to this article.

^1^State Key Laboratory for Diagnosis and Treatment of Infectious Diseases, National Clinical Research Center for Infectious Diseases, Collaborative Innovation Center for Diagnosis and Treatment of Infectious Diseases, Department of Infectious Diseases, The First Affiliated Hospital, College of Medicine, Zhejiang University

^2^Department of Gastroenterology, the First Affiliated Hospital, College of Medicine, Zhejiang University

^3^Key Laboratory of Combined Multi-Organ Transplantation Division of Hepatobiliary and Pancreatic Surgery, the First Affiliated Hospital, School of Medicine, Zhejiang University.

*Address correspondence to Yida Yang, Jifang Sheng, Tingbo Liang and Lanjuan Li. The First Affiliated Hospital, College of Medicine, Zhejiang University, 79 Qingchun Rd., Hangzhou City 310003, China (yangyida65@163.com; jifang_sheng@zju.edu.cn; liangtingbo@zju.edu.cn; ljli@zju.edu.cn; 86-0571-87236749)

**Table of Contents**

**Supplemental Table 1:** Univariate Analysis of Risk Factors for the 49 severe/Critical type Patients. P4

**ZJ01 sequence**  P5-19

**Supplemental Table1**. Univariate Analysis of Risk Factors for the 49 severe/Critical type Patients

| Risk Factor | Odds Ratio  (95% CI) | p Value |
| --- | --- | --- |
| Age | 1.044(1.023-1.065) | 0.000 |
| Age ≥ 50 yr | 2.283（1.229-4.240） | 0.009 |
| Sex(Femal) | 0.445(0.236-0.842) | 0.013 |
| Any Coexisting Disease | 5.505（2.937-10.318） | 0.000 |
| Hypertesion | 4.365（2.330-8.176） | 0.000 |
| Cough | 2.742(1.252-6.005) | 0.012 |
| Sputum | 2.065(1.137-3.752) | 0.017 |
| Hemoptysis | 11.705（3.031-45.196） | 0.000 |
| Muscle ache | 3.135(1.511-6.504) | 0.002 |
| Gastrointestinal symptoms | 3.491（1.708-7.134） | 0.001 |
| Diarrhea | 4.527（2.068-9.910） | 0.000 |
| Headache | 2.410（1.119-5.192） | 0.025 |
| Lymphocytes | 0.104（0.043-0.252） | 0.000 |
| Decreased Lymphocytes | 3.445（1.813-6.547） | 0.000 |
| Increased Neutrophils | 4.614（1.509-14.107） | 0.007 |
| C-reactive protein | 1.035(1.023-1.047) | 0.000 |
| Albumin | 0.808(0.748-0.873) | 0.000 |
| Alanine aminotransferase | 1.011(1.001-1.021) | 0.035 |
| Aspartate aminotransferase | 1.018(1.006-1.031) | 0.004 |
| Blood urea nitrogen | 1.213(1.046-1.406) | 0.011 |
| Serum creatinine | 1.016(1.004-1.028) | 0.009 |
| Serum sodium | 0.913(0.845-0.988) | 0.023 |
| Glucose | 1.22(1.075-1.385) | 0.002 |
| Creatine kinase | 1.002(1.001-1.003) | 0.004 |
| Lactate dehydrogenase | 1.001(1.000-1.003) | 0.027 |

**Appendix-ZJ01 sequence**

>BataCov/Zhejiang/ZJ01/2019

TAAAGGTTTATACCTTCCCAGGTAACAAACCAACCAACTTTCGATCTCTT

GTAGATCTGTTCTCTAAACGAACTTTAAAATCTGTGTGGCTGTCACTCGG

CTGCATGCTTAGTGCACTCACGCAGTATAATTAATAACTAATTACTGTCG

TTGACAGGACACGAGTAACTCGTCTATCTTCTGCAGGCTGCTTACGGTTT

CGTCCGTGTTGCAGCCGATCATCAGCACATCTAGGTTTCGTCCGGGTGTG

ACCGAAAGGTAAGATGGAGAGCCTTGTCCCTGGTTTCAACGAGAAAACAC

ACGTCCAACTCAGTTTGCCTGTTTTACAGGTTCGCGACGTGCTCGTACGT

GGCTTTGGAGACTCCGTGGAGGAGGTCTTATCAGAGGCACGTCAACATCT

TAAAGATGGCACTTGTGGCTTAGTAGAAGTTGAAAAAGGCGTTTTGCCTC

AACTTGAACAGCCCTATGTGTTCATCAAACGTTCGGATGCTCGAACTGCA

CCTCATGGTCATGTTATGGTTGAGCTGGTAGCAGAACTCGAAGGCATTCA

GTACGGTCGTAGTGGTGAGACACTTGGTGTCCTTGTCCCTCATGTGGGCG

AAATACCAGTGGCTTACCGCAAGGTTCTTCTTCGTAAGAACGGTAATAAA

GGAGCTGGTGGCCATAGTTACGGCGCCGATCTAAAGTCATTTGACTTAGG

CGACGAGCTTGGCACTGATCCTTATGAAGATTTTCAAGAAAACTGGAACA

CTAAACATAGCAGTGGTGTTACCCGTGAACTCATGCGTGAGCTTAACGGA

GGGGCATACACTCGCTATGTCGATAACAACTTCTGTGGCCCTGATGGCTA

CCCTCTTGAGTGCATTAAAGACCTTCTAGCACGTGCTGGTAAAGCTTCAT

GCACTTTGTCCGAACAACTGGACTTTATTGACACTAAGAGGGGTGTATAC

TGCTGCCGTGAACATGAGCATGAAATTGCTTGGTACACGGAACGTTCTGA

AAAGAGCTATGAATTGCAGACACCTTTTGAAATTAAATTGGCAAAGAAAT

TTGACACCTTCAATGGGGAATGTCCAAATTTTGTATTTCCCTTAAATTCC

ATAATCAAGACTATTCAACCAAGGGTTGAAAAGAAAAAGCTTGATGGCTT

TATGGGTAGAATTCGATCTGTCTATCCAGTTGCGTCACCAAATGAATGCA

ACCAAATGTGCCTTTCAACTCTCATGAAGTGTGATCATTGTGGTGAAACT

TCATGGCAGACGGGCGATTTTGTTAAAGCCACTTGCGAATTTTGTGGCAC

TGAGAATTTGACTAAAGAAGGTGCCACTACTTGTGGTTACTTACCCCAAA

ATGCTGTTGTTAAAATTTATTGTCCAGCATGTCACAATTCAGAAGTAGGA

CCTGAGCATAGTCTTGCCGAATACCATAATGAATCTGGCTTGAAAACCAT

TCTTCGTAAGGGTGGTCGCACTATTGCCTTTGGAGGCTGTGTGTTCTCTT

ATGTTGGTTGCCATAACAAGTGTGCCTATTGGGTTCCACGTGCTAGCGCT

AACATAGGTTGTAACCATACAGGTGTTGTTGGAGAAGGTTCCGAAGGTCT

TAATGACAACCTTCTTGAAATACTCCAAAAAGAGAAAGTCAACATCAATA

TTGTTGGTGACTTTAAACTTAATGAAGAGATCGCCATTATTTTGGCATCT

TTTTCTGCTTCCACAAGTGCTTTTGTGGAAACTGTGAAAGGTTTGGATTA

TAAAGCATTCAAACAAATTGTTGAATCCTGTGGTAATTTTAAAGTTACAA

AAGGAAAAGCTAAAAAAGGTGCCTGGAATATTGGTGAACAGAAATCAATA

CTGAGTCCTCTTTATGCATTTGCATCAGAGGCTGCTCGTGTTGTACGATC

AATTTTCTCCCGCACTCTTGAAACTGCTCAAAATTCTGTGCGTGTTTTAC

AGAAGGCCGCTATAACAATACTAGATGGAATTTCACAGTATTCACTGAGA

CTCATTGATGCTATGATGTTCACATCTGATTTGGCTACTAACAATCTAGT

TGTAATGGCCTACATTACAGGTGGTGTTGTTCAGTTGACTTCGCAGTGGC

TAACTAACATCTTTGGCACTGTTTATGAAAAACTCAAACCCGTCCTTGAT

TGGCTTGAAGAGAAGTTTAAGGAAGGTGTAGAGTTTCTTAGAGACGGTTG

GGAAATTGTTAAATTTATCTCAACCTGTGCTTGTGAAATTGTCGGTGGAC

AAATTGTCACCTGTGCAAAGGAAATTAAGGAGAGTGTTCAGACATTCTTT

AAGCTTGTAAATAAATTTTTGGCTTTGTGTGCTGACTCTATCATTATTGG

TGGAGCTAAACTTAAAGCCTTGAATTTAGGTGAAACATTTGTCACGCACT

CAAAGGGATTGTACAGAAAGTGTGTTAAATCCAGAGAAGAAACTGGCCTA

CTCATGCCTCTAAAAGCCCCAAAAGAAATTATCTTCTTAGAGGGAGAAAC

ACTTCCCACAGAAGTGTTAACAGAGGAAGTTGTCTTGAAAACTGGTGATT

TACAACCATTAGAACAACCTACTAGTGAAGCTGTTGAAGCTCCATTGGTT

GGTACACCAGTTTGTATTAACGGGCTTATGTTGCTCGAAATCAAAGACAC

AGAAAAGTACTGTGCCCTTGCACCTAATATGATGGTAACAAACAATACCT

TCACACTCAAAGGCGGTGCACCAACAAAGGTTACTTTTGGTGATGACACT

GTGATAGAAGTGCAAGGTTACAAGAGTGTGAATATCACTTTTGAACTTGA

TGAAAGGATTGATAAAGTACTTAATGAGAAGTGCTCTGCCTATACAGTTG

AACTCGGTACAGAAGTAAATGAGTTCGCCTGTGTTGTGGCAGATGCTGTC

ATAAAAACTTTGCAACCAGTATCTGAATTACTTACACCACTGGGCATTGA

TTTAGATGAGTGGAGTATGGCTACATACTACTTATTTGATGAGTCTGGTG

AGTTTAAATTGGCTTCACATATGTATTGTTCTTTCTACCCTCCAGATGAG

GATGAAGAAGAAGGTGATTGTGAAGAAGAAGAGTTTGAGCCATCAACTCA

ATATGAGTATGGTACTGAAGATGATTACCAAGGTAAACCTTTGGAATTTG

GTGCCACTTCTGCTGCTCTTCAACCTGAAGAAGAGCAAGAAGAAGATTGG

TTAGATGATGATAGTCAACAAACTGTTGGTCAACAAGACGGCAGTGAGGA

CAATCAGACAACTACTATTCAAACAATTGTTGAGGTTCAACCTCAATTAG

AGATGGAACTTACACCAGTTGTTCAGACTATTGAAGTGAATAGTTTTAGT

GGTTATTTAAAACTTACTGACAATGTATACATTAAAAATGCAGACATTGT

GGAAGAAGCTAAAAAGGTAAAACCAACAGTGGTTGTTAATGCAGCCAATG

TTTACCTTAAACATGGAGGAGGTGTTGCAGGAGCCTTAAATAAGGCTACT

AACAATGCCATGCAAGTTGAATCTGATGATTACATAGCTACTATGGACCA

CTTAAAGTGGGTGGTAGTTGTGTTTTAAGCGGACACAATCTTGCTAAACA

CTGTCTTCATGTTGTCGGCCCAAATGTTAACAAAGGTGAAGACATTCAAC

TTCTTAAGAGTGCTTATGAAAATTTTAATCAGCACGAAGTTCTACTTGCA

CCATTATTATCAGCTGGTATTTTTGGTGCTGACCCTATACATTCTTTAAG

AGTTTGTGTAGATACTGTTCGCACAAATGTCTACTTAGCTGTCTTTGATA

AAAATCTCTATGACAAACTTGTTTCAAGCTTTTTGGAAATGAAGAGTGAA

AAGCAAGTTGAACAAAAGATCGCTGAGATTCCTAAAGAGGAAGTTAAGCC

ATTTATAACTGAAAGTAAACCTTCAGTTGAACAGAGAAAACAAGATGATA

AGAAAATCAAAGCTTGTGTTGAAGAAGTTACAACAACTCTGGAAGAAACT

AAGTTCCTCACAGAAAACTTGTTACTTTATATTGACATTAATGGCAATCT

TCATCCAGATTCTGCCACTCTTGTTAGTGACATTGACATCACTTTCTTAA

AGAAAGATGCTCCATATATAGTGGGTGATGTTGTTCAAGAGGGTGTTTTA

ACTGCTGTGGTTATACCTACTAAAAAGGCTGGTGGCACTACTGAAATGCT

AGCGAAAGCTTTGAGAAAAGTGCCAACAGACAATTATATAACCACTTACC

CGGGTCAGGGTTTAAATGGTTACACTGTAGAGGAGGCAAAGACAGTGCTT

AAAAAGTGTAAAAGTGCCTTTTACATTCTACCATCTATTATCTCTAATGA

GAAGCAAGAAATTCTTGGAACTGTTTCTTGGAATTTGCGAGAAATGCTTG

CACATGCAGAAGAAACACGCAAATTAATGCCTGTCTGTGTGGAAACTAAA

GCCATAGTTTCAACTATACAGCGTAAATATAAGGGTATTAAAATACAAGA

GGGTGTGGTTGATTATGGTGCTAGATTTTACTTTTACACCAGTAAAACAA

CTGTAGCGTCACTTATCAACACACTTAACGATCTAAATGAAACTCTTGTT

ACAATGCCACTTGGCTATGTAACACATGGCTTAAATTTGGAAGAAGCTGC

TCGGTATATGAGATCTCTCAAAGTGCCAGCTACAGTTTCTGTTTCTTCAC

CTGATGCTGTTACAGCGTATAATGGTTATCTTACTTCTTCTTCTAAAACA

CCTGAAGAACATTTTATTGAAACCATCTCACTTGCTGGTTCCTATAAAGA

TTGGTCCTATTCTGGACAATCTACACAACTAGGTATAGAATTTCTTAAGA

GAGGTGATAAAAGTGTATATTACACTAGTAATCCTACCACATTCACCTAG

ATGTGAAGTTATCACCTTTGACAATCTTAAGACACTTCTTTCTTTGAGAG

AAGTGAGGACTATTAAGGTGTTTACAACAGTAGACAACATTAACCTCCAC

ACGCAAGTTGTGGACATGTCAATGACATATGGACAACAGTTTGGTCCAAC

TTATTTGGATGGAGCTGATGTTACTAAAATAAAACCTCATAATTCACATG

AAGGTAAAACATTTTATGTTTTACCTAATGATGACACTCTACGTGTTGAG

GCTTTTGAGTACTACCACACAACTGATCCTAGTTTTCTGGGTAGGTACAT

GTCAGCATTAAATCACACTAAAAAGTGGAAATACCCACAAGTTAATGGTT

TAACTTCTATTAAATGGGCAGATAACAACTGTTATCTTGCCACTGCATTG

TTAACACTCCAACAAATAGAGTTGAAGTTTAATCCACCTGCTCTACAAGA

TGCTTATTACAGAGCAAGGGCTGGTGAAGCTGCTAACTTTTGTGCACTTA

TCTTAGCCTACTGTAATAAGACAGTAGGTGAGTTAGGTGATGTTAGAGAA

ACAATGAGTTACTTGTTTCAACATGCCAATTTAGATTCTTGCAAAAGAGT

CTTGAACGTGGTGTGTAAAACTTGTGGACAACAGCAGACAACCCTTAAGG

GTGTAGAAGCTGTTATGTACATGGGCACACTTTCTTATGAACAATTTAAG

AAAGGTGTTCAGATACCTTGTACGTGTGGTAAACAAGCTACAAAATATCT

AGTACAACAGGAGTCACCTTTTGTTATGATGTCAGCACCACCTGCTCAGT

ATGAACTTAAGCATGGTACATTTACTTGTGCTAGTGAGTACACTGGTAAT

TACCAGTGTGGTCACTATAAACATATAACTTCTAAAGAAACTTTGTATTG

CATAGACGGTGCTTTACTTACAAAGTCCTCAGAATACAAAGGTCCTATTA

CGGATGTTTTCTACAAAGAAAACAGTTACACAACAACCATAAAACCAGTT

ACTTATAAATTGGATGGTGTTGTTTGTACAGAAATTGACCCTAAGTTGGA

CAATTATTATAAGAAAGACAATTCTTATTTCACAGAGCAACCAATTGATC

TTGTACCAAACCAACCATATCCAAACGCAAGCTTCGATAATTTTAAGTTT

GTATGTGATAATATCAAATTTGCTGATGATTTAAACCAGTTAACTGGTTA

TAAGAAACCTGCTTCAAGAGAGCTTAAAGTTACATTTTTCCCTGACTTAA

ATGGTGATGTGGTGGCTATTGATTATAAACACTACACACCCTCTTTTAAG

AAAGGAGCTAAATTGTTACATAAACCTATTGTTTGGCATGTTAACAATGC

AACTAATAAAGCCACGTATAAACCAAATACCTGGTGTATACGTTGTCTTT

GGAGCACAAAACCAGTTGAAACATCAAATTCGTTTGATGTACTGAAGTCA

GAGGACGCGCAGGGAATGGATAATCTTGCCTGCGAAGATCTAAAACCAGT

CTCTGAAGAAGTAGTGGAAAATCCTACCATACAGAAAGACGTTCTTGAGT

GTAATGTGAAAACTACCGAAGTTGTAGGAGACATTATACTTAAACCAGCA

AATAATAGTTTAAAAATTACAGAAGAGGTTGGCCACACAGATCTAATGGC

TGCTTATGTAGACATTCTAGTCTTACTATTAAGAAACCTAATGAATTATC

TAGAGTATTAGGTTTGAAAACCCTTGCTACTCATGGTTTAGCTGCTGTTA

ATAGTGTCCCTTGGGATACTATAGCTAATTATGCTAAGCCTTTTCTTAAC

AAAGTTGTTAGTACAACTACTAACATAGTTACACGGTGTTTAAACCGTGT

TTGTACTAATTATATGCCTTATTTCTTTACTTTATTGCTACAATTGTGTA

CTTTTACTAGAAGTACAAATTCTAGAATTAAAGCATCAATCCCGACTACC

ATGGCAAAGAATACTGTTAAGAGTGTCGGTTAATTTTGTCTACAGGCTTC

ATTTAATTATTTGAAGTCACCTAATTTTTCTAAACTGATAAATATTATAA

TTTGGTTTTTACTATTAAGTGTTTGCCTAGGTTCTTTAATCTACTCAACC

GCTGCTTTAGGTGTTTTAATGTCTAATTTAGGCATGCCTTCTTACTGTAC

TGGATACAGAGAAGGCTATTTGAACTCTACTAATGTCACTATTGCAACCT

ACTGTACTGGTTCTATACCTTGTAGTGTTTGTCTTAGTGGTTTAGATTCT

TTAGACACCTATCCTTCTTTAGAAACTATACAAATTACCATTTCATCTTT

TAAATGGGATTTAACTGCTTTTGGCTTAGTTGCAGAGTGGTTTTTGGCAT

ATATTCTTTTCACTAGGTTTTTCTATGTACTTGGATTGGCTGCAATCATG

CAATTGTTTTTCAGCTATTTTGCAGTACATTTTATTAGTAATTCTTGGCT

TATGTGGTTAATAATTAATCTTGTACAAATGGCCCCGATTTCAGCTATGG

TTAGAATGTACATCTTCTTTGCATCATTTTATTATGTATGGAAAAGTTAT

GTGCATGTTGTAGACGGTTGTAATTCATCAACTTGTATGATGTGTTACAA

ACGTAATAGAGCAACAAGAGTCGAATGTACACCTATTGTTAATGGTGTTA

GAAGGTCCTTTTATGTCTATGATAATGGAGGTAAAGGCTTTTGCAAACTA

CACAATTGGAATTGTGTTAATTGTGATACATTCTGTGCTGGTAGTACATT

TATTAGTGATGAAGTTGCGAGAGACTTGTCACTACAGTTTAAAAGACCAA

TAAATCCTACTGACCAGTCTTCTTACATCGTTGATAGTGTTACAGTGAAG

AATGGTTCCATCCATCTTTACTTTGATAAAGCTGGTCAAAAGACTTATGA

AAGACATTCTCTCTCTCATTTTGTTAACTTAGACAACCTGAGAGCTAATA

ACACTAAAGGTTCATTGCCTATTAATGTTATAGTTTTTGATGGTAAATCA

AAATGTGAAGAATCATCTGCAAAATCAGCGTCTGTTTACTACAGTCAGCT

TATGTGTCAACCTATACTGTTACTAGATCAGGCATTAGTGTCTGATGTTG

GTGATAGTGCGGAAGTTGCAGTTAAAATGTTTGATGCTTACGTTAATACG

TTTTCATCAACTTTTAACGTACCAATGGAAAAACTCAAAACACTAGTTGC

AACTGCAGAAGCTGAACTTGCAAAGAATGTGTCCTTAGACAATGTCTTAT

CTACTTTTATTTCAGCAGCTCGGCAAGGGTTTGTTGATTCAGATGTAGAA

ACTAAAGATGTTGTTGAATGTCTTAAATTGTCACATCAATCTGACATAGA

AGTTACTGGCGATAGTTGTAATAACTATATGCTCACCTATAACAAAGTTG

AAAACATGACACCCCGTGACCTTGGTGCTTGTATTGACTGTAGTGCGCGT

CATATTAATGCGCAGGTAGCAAAAAGTCACAACATTGCTTTGATATGGAA

CGTTAAAGATTTCATGTCATTGTCTGAACAACTACGAAAACAAATACGTA

GTGCTGCTAAAAAGAATAACTTACCTTTTAAGTTGACATGTGCAACTACT

AGACAAGTTGTTAATGTTGTAACAACAAAGATAGCACTTAAGGGTGGTAA

AATTGTTAATAATTGGTTGAAGCAGTTAATTAAAGTTACACTTGTGTTCC

TTTTTGTTGCTGCTATTTTCTATTTAATAACACCTGTTCATGTCATGTCT

AAACATACTGACTTTTCAAGTGAAATCATAGGATACAAGGCTATTGATGG

TGGTGTCACTCGTGACATAGCATCTACAGATACTTGTTTTGCTAACAAAC

ATGCTGATTTTGACACATGGTTTAGTCAGCGTGGTGGTAGTTATACTAAT

GACAAAGCTTGCCCATTGATTGCTGCAGTCATAACAAGAGAAGTGGGTTT

TGTCGTGCCTGGTTTGCCTGGCACGATATTACGCACAACTAATGGTGACT

TTTTGCATTTCTTACCTAGAGTTTTTAGTGCAGTTGGTAACATCTGTTAC

ACACCATCAAAACTTATAGAGTACACTGACTTTGCAACATCAGCTTGTGT

TTTGGCTGCTGAATGTACAATTTTTAAAGATGCTTCTGGTAAGCCAGTAC

CATATTGTTATGATACCAATGTACTAGAAGGTTCTGTTGCTTATGAAAGT

TTACGCCCTGACACACGTTATGTGCTCATGGATGGCTCTATTATTCAATT

TCCTAACACCTACCTTGAAGGTTCTGTTAGAGTGGTAACAACTTTTGATT

CTGAGTACTGTAGGCACGGCACTTGTGAAAGATCAGAAGCTGGTGTTTGT

GTATCTACTAGTGGTAGATGGGTACTTAACAATGATTATTACAGATCTTT

ACCAGGAGTTTTCTGTGGTGTAGATGCTGTAAATTTACTTACTAATATGT

TTACACCACTAATTCAACCTATTGGTGCTTTGGACATATCAGCATCTATA

GTAGCTGGTGGTATTGTAGCTATCGTAGTAACATGCCTTGCCTACTATTT

TATGAGGTTTAGAAGAGCTTTTGGTGAATACAGTCATGTAGTTGCCTTTA

ATACTTTACTATTCCTTATGTCATTCACTGTACTCTGTTTAACACCAGTT

TACTCATTCTTACCTGGTGTTTATTCTGTTATTTACTTGTACTTGACATT

TTATCTTACTAATGATGTTTCTTTTTTAGCACATATTCAGTGGATGGTTA

TGTTCACACCTTTAGTACCTTTCTGGATAACAATTGCTTATATCATTTGT

ATTTCCACAAAGCATTTCTATTGGTTCTTTAGTAATTACCTAAAGAGACG

TGTAGTCTTTAATGGTGTTTCCTTTAGTACTTTTGAAGAAGCTGCGCTGT

GCACCTTTTTGTTAAATAAAGAAATGTATCTAAAGTTGCGTAGTGATGTG

CTATTACCTCTTACGCAATATAATAGATACTTAGCTCTTTATAATAAGTA

CAAGTATTTTAGTGGAGCAATGGATACAACTAGCTACAGAGAAGCTGCTT

GTTGTCATCTCGCAAAGGCTCTCAATGACTTCAGTAACTCAGGTTCTGAT

GTTCTTTACCAACCACCACAAACCTCTATCACCTCAGCTGTTTTGCAGAG

TGGTTTTAGAAAAATGGCATTCCCATCTGGTAAAGTTGAGGGTTGTATGG

TACAAGTAACTTGTGGTACAACTACACTTAACGGTCTTTGGCTTGATGAC

GTAGTTTACTGTCCAAGACATGTGATCTGCACCTCTGAAGACATGCTTAA

CCCTAATTATGAAGATTTACTCATTCGTAAGTCTAATCATAATTTCTTGG

TACAGGCTGGTAATGTTCAACTCAGGGTTATTGGACATTCTATGCAAAAT

TGTGTACTTAAGCTTAAGGTTGATACAGCCAATCCTAAGACACCTAAGTA

TAAGTTTGTTCGCATTCAACCAGGACAGACTTTTTCAGTGTTAGCTTGTT

ACAATGGTTCACCATCTGGTGTTTACCAATGTGCTATGAGGCCCAATTTC

ACTATTAAGGGTTCATTCCTTAATGGTTCATGTGGTAGTGTTGGTTTTAA

CATAGATTATGACTGTGTCTCTTTTTGTTACATGCACCATATGGAATTAC

CAACTGGAGTTCATGCTGGCACAGACTTAGAAGGTAACTTTTATGGACCT

TTTGTTGACAGGCAAACAGCACAAGCAGCTGGTACGGACACAACTATTAC

AGTTAATGTTTTAGCTTGGTTGTACGCTGCTGTTATAAATGGAGACAGGT

GGTTTCTCAATCGATTTACCACAACTCTTAATGACTTTAACCTTGTGGCT

ATGAAGTACAATTATGAACCTCTAACACAAGACCATGTTGACATACTAGG

ACCTCTTTCTGCTCAAACTGGAATTGCCGTTTTAGATATGTGTGCTTCAT

TAAAAGAATTACTGCAAAATGGTATGAATGGACGTACCATATTGGGTAGT

GCTTTATTAGAAGATGAATTTACACCTTTTGATGTTGTTAGACAATGCTC

AGGTGTTACTTTCCAAAGTGCAGTGAAAAGAACAATCAAGGGTACACACC

ACTGGTTGTTACTCACAATTTTGACTTCACTTTTAGTTTTAGTCCAGAGT

ACTCAATGGTCTTTGTTCTTTTTTTTGTATGAAAATGCCTTTTTACCTTT

TGCTATGGGTATTATTGCTATGTCTGCTTTTGCAATGATGTTTGTCAAAC

ATAAGCATGCATTTCTCTGTTTGTTTTTGTTACCTTCTCTTGCCACTGTA

GCTTATTTTAATATGGTCTATATGCCTGCTAGTTGGGTGATGCGTATTAT

GACATGGTTGGATATGGTTGATACTAGTTTGTCTGGTTTTAAGCTAAAAG

ACTGTGTTATGTATGCATCAGCTGTAGTGTTACTAATCCTTATGACAGCA

AGAACTGTGTATGATGATGGTGCTAGGAGAGTGTGGACACTTATGAATGT

CTTGACACTCGTTTATAAAGTTTATTATGGTAATGCTTTAGATCAAGCCA

TTTCCATGTGGGCTCTTATAATCTCTGTTACTTCTAACTACTCAGGTGTA

GTTACAACTGTCATGTTTTTGGCCAGAGGTATTGTTTTTATGTGTGTTGA

GTATTGCCCTATTTTCTTCATAACTGGTAATACACTTCAGTGTATAATGC

TAGTTTATTGTTTCTTAGGCTATTTTTGTACTTGTTACTTTGGCCTCTTT

TGTTTACTCAACCGCTACTTTAGACTGACTCTTGGTGTTTATGATTACTT

AGTTTCTACACAGGAGTTTAGATATATGAATTCACAGGGACTACTCCCAC

CCAAGAATAGCATAGATGCCTTCAAACTCAACATTAAATTGTTGGGTGTT

GGTGGCAAACCTTGTATCAAAGTAGCCACTGTACAGTCTAAAATGTCAGA

TGTAAAGTGCACATCAGTAGTCTTACTCTCAGTTTTGCAACAACTCAGAG

TAGAATCATCATCTAAATTGTGGGCTCAATGTGTCCAGTTACACAATGAC

ATTCTCTTAGCTAAAGATACTACTGAAGCCTTTGAAAAAATGGTTTCACT

ACTTTCTGTTTTGCTTTCCATGCAGGGTGCTGTAGACATAAACAAGCTTT

GTGAAGAAATGCTGGACAACAGGGCAACCTTACAAGCTATAGCCTCAGAG

TTTAGTTCCCTTCCATCATATGCAGCTTTTGCTACTGCTCAAGAAGCTTA

TGAGCAGGCTGTTGCTAATGGTGATTCTGAAGTTGTTCTTAAAAAGTTGA

AGAAGTCTTTGAATGTGGCTAAATCTGAATTTGACCGTGATGCAGCCATG

CAACGTAAGTTGGAAAAGATGGCTGATCAAGCTATGACCCAAATGTATAA

ACAGGCTAGATCTGAGGACAAGAGGGCAAAAGTTACTAGTGCTATGCAGA

CAATGCTTTTCACTATGCTTAGAAAGTTGGATAATGATGCACTCAACAAC

ATTATCAACAATGCAAGAGATGGTTGTGTTCCCTTGAACATAATACCTCT

TACAACAGCAGCCAAACTAATGGTTGTCATACCAGACTATAACACATATA

AAAATACGTGTGATGGTACAACATTACTTATGCATCAGCATTGTGGGAAA

TCCAACAGGTTGTAGATGCAGATAGTAAAATTGTTCAACTTAGTGAAATT

AGTATGGACAATTCACCTAATTTAGCATGGCCTCTTATTGTAACAGCTTT

AAGGGCCAATTCTGCTGTCAAATTACAGAATAATGAGCTTAGTCCTGTTG

CACTACGACAGATGTCTTGTGCTGCCGGTACTACACAAACTGCTTGCACT

GATGACAATGCGTTAGCTTACTACAACACAACAAAGGGAGGTAGGTTTGT

ACTTGCACTGTTATCCGATTTACAGGATTTGAAATGGGCTAGATTCCCTA

AGAGTGATGGAACTGGTACTATCTATACAGAACTGGAACCACCTTGTAGG

TTTGTTACAGACACACCTAAAGGTCCTAAAGTGAAGTATTTATACTTTAT

TAAAGGATTAAACAACCTAAATAGAGGTATGGTACTTGGTAGTTTAGCTG

CCACAGTACGTCTACAAGCTGGTAATGCAACAGAAGTGCCTGCCAATTCA

ACTGTATTATCTTTCTGTGCTTTTGCTGTAGATGCTGCTAAAGCTTACAA

AGATTATCTAGCTAGTGGGGGACAACCAATCACTAATTTGTGTTAAGATG

TTGTGTACACAACACTGGTACTGGTCAGGCAAATAACAGTTACACCGGAA

GCCAATATGGATCAAAGAATCCTTTGGTGGTGCATCGTGTTGTCTGTACT

GCCGTTGCCACATAGATCATCCAAATCCTAAAGGATTTTGTGACTTAAAA

GGTAAGTATGTACAAATACCTACAACTTGTGCTAATGACCCTGTGGGTTT

TACACTTAAAAACACAGTCTGTACCGTCTGCGGTATGTGGAAAGGTTATG

GCTGTAGTTGTGATCAACTCCGCGAACCCATGCTTCAGTCAGCTGATGCA

CAATCGTTTTTAAACGGGTTTGCGGTGTAAGTGCAGCCCGTCTTACACCG

TGCGGCACAGGCACTAGTACTGATGTCGTATACAGGGCTTTTGACATCTA

CAATGATAAAGTAGCTGGTTTTGCTAAATTCCTAAAAACTAATTGTTGTC

GCTTCCAAGAAAAGGACGAAGATGACAATTTAATTGATTCTTACTTTGTA

GTTAAGAGACACACTTTCTCTAACTACCAACATGAAGAAACAATTTATAA

TTTACTTAAGGATTGTCCAGCTGTTGCTAAACATGACTTCTTTAAGTTTA

GAATAGACGGTGACATGGTACCACATATATCACGTCAACGTCTTACTAAA

TACACAATGGCAGACCTCGTCTATGCTTTAAGGCATTTTGATGAAGGTAA

TTGTGACACATTAAAAGAAATACTTGTCACATACAATTGTTGTGATGATG

ATTATTTCAATAAAAAGGACTGGTATGATTGAGAAAAATATTTGTTGATG

GTGTTCCATTTGTAGTTTCAACTGGATACCACTTCAGAGAGCTAGGTGTT

GTACATAATCAGGATGTAAACTTACATAGCTCTAGACTTAGTTTTAAGGA

ATTACTTGTGTATGCTGCTGACCCTGCTATGCACGCTGCTTCTGGTAATC

TATTACTAGATAAACGCACTACGTGCTTTTCAGTAGCTGCACTTACTAAC

AATGTTGCTTTTCAAACTGTCAAACCCGGTAATTTTAACAAAGACTTCTA

TGACTTTGCTGTGTCTAAGGGTTTCTTTAAGGAAGGAAGTTCTGTTGAAT

TAAAACACTTCTTCTTTGCTCAGGATGGTAATGCTGCTATCAGCGATTAT

GACTACTATCGTTATAATCTACCAACAATGTGTGATATCAGACAACTACT

ATTTGTAGTTGAAGTTGTTGATAAGTACTTTGATTGTTACGATGGTGGCT

GTATTAATGCTAACCAAGTCATCGTCAACAACCTAGACAAATCAGCTGGT

TTTCCATTTAATAAATGGGGTAAGGCTAGACTTTATTATGATTCAATGAG

TTATGAGGATCAAGATGCACTTTTCGCATATACAAAACGTAATGTCATCC

CTACTATAACTCAAATGAATCTTAAGTATGCCATTAGTGCAAAGAATAGA

GCTCGCACCGTAGCTGGTGTCTCTATCTGTAGTACTATGACCAATAGACA

GTTTCATCAAAAATTATTGAAATCAATAGCCGCCACTAGAGGAGCTACTG

TAGTAATTGGAACAAGCAAATTCTATGGTGGTTGGCACAACATGTTAAAA

ACTGTTTATAGTGATGTAGAAAACCCTCACCTTATGGGTTGGGATTATCC

TAAATGTGATAGAGCCATGCCTAACATGCTTAGAATTATGGCCTCACTTG

TTCTTGCTCGCAAACATACAACGTGTTGTAGCTTGTCACACCGTTTCTAT

AGATTAGCTAATGAGTGTGCTCAAGTATTGAGTGAAATGGTCATGTGTGG

CGGTTCACTATATGTTAAACCAGGTGGGACCTCATCAGGAGATGCCACAA

CTGCTTATGCTAATAGTGTTTTTAACATTTGTCAAGCTGTCACGGCCAAT

GTTAATGCACTTTTATCTACTGATGGTAACAAAATTGCCGATAAGTATGT

CCGCAATTTACAACACAGACTTTATGAGTGTCTCTATAGAAATAGAGATG

TTGACACAGACTTTGTGAATGAGTTTTACGCATATTTGCGTAAACATTTC

TCAATGATGATACTCTCTGACGATGCTGTTGTGTGTTTCAATAGCACTTA

TGCATCTCAAGGTCTAGTGGCTAGCATAAAGAACTTTAAGTCAGTTCTTT

ATTATCAAAACAATGTTTTTATGTCTGAAGCAAAATGTTGGACTGAGACT

GACCTTACTAAAGGACCTCATGAATTTTGCTCTCAACATACAATGCTAGT

TAAACAGGGTGATGATTATGTGTACCTTCCTTACCCAGATCCATCAAGAA

TCCTAGGGGCCGGCTGTTTTGTAGATGATATCGTAAAAACAGATGGTACA

CTTATGATTGAACGGTTCGTGTCTTTAGCTATAGATGCTTACCCACTTAC

TAAACATCCTAATCAGGAGTATGCTGATGTCTTTCATTTGTACTTACAAT

ACATAAGAAAGCTACATGATGAGTTAACAGGACACATGTTAGACATGTAT

TCTGTTATGCTTACTAATGATAACACTTCAAGGTATTGGGAACCTGAGTT

TTATGAGGCTATGTACACACCGCATACAGTCTTACAGGCTGTTGGGGCTT

GTGTTCTTTGCAATTCACAGACTTCATTAAGATGTGGTGCTTGCATACGT

AGACCATTCTTATGTTGTAAATGCTGTTACGACCATGTCATATCAACATC

ACATAAATTAGTCTTGTCTGTTAATCCGTATGTTTGCAATGCTCCAGGTT

GTGATGTCACAGATGTGACTCAACTTTACTTAGGAGGTATGAGCTATTAT

TGTAAATCACATAAACCACCCATTAGTTTTCCATTGTGTGCTAATGGACA

AGTTTTTGGTTTATATAAAAATACATGTGTTGGTAGCGATAATGTTACTG

ACTTTAATGCAATTGCAACATGTGACTGGACAAATGCTGGTGATTACATT

TTAGCTAACACCTGTACTGAAAGACTCAAGCTTTTTGCAGCAGAAACGCT

CAAAGCTACTGAGGAGACATTTAAACTGTCTTATGGTATTGCTACTGTAC

GTGAAGTGCTGTCTGACAGAGAATTACATCTTTCATGGGAAGTTGGTAAA

CCTAGACCACCACTTAACCGAAATTATGTCTTTACTGGTTATCGTGTAAC

TAAAAACAGTAAAGTACAAATAGGAGAGTACACCTTTGAAAAAGGTGACT

ATGGTGATGCTGTTGTTTACCGAGGTACAACAACTTACAAATTAAATGTT

GGTGATTATTTTGTGCTGACATCACATACAGTAATGCCATTAAGTGCACC

TACACTAGTGCCACAAGAGCACTATGTTAGAATTACTGGCTTATACCCAA

CACTCAATATCTCAGATGAGTTTTCTAGCAATGTTGCAAATTATCAAAAG

GTTGGTATGCAAAAGTATTCTACACTCCAGGGACCACCTGGTACTGGTAA

GAGTCATTTTGCTATTGGCCTAGCTCTCTACTACCCTTCTGCTCGCATAG

TGTATACAGCTTGCTCTCATGCCGCTGTTGATGCACTATGTGAGAAGGCA

TTAAAATATTTGCCTATAGATAAATGTAGTAGAATTATACCTGCACGTGC

TCGTGTAGAGTGTTTTGATAAATTCAAAGTGAATTCAACATTAGAACAGT

ATGTCTTTTGTACTGTAAATGCATTGCCTGAGACGACAGCAGATATAGTT

GTCTTTGATGAAATTTCAATGGCCACAAATTATGATTTGAGTGTTGTCAA

TGCCAGATTACGTGCTAAGCACTATGTGTACATTGGCGACCCTGCTCAAT

TACCTGCACCACGCACATTGCTAACTAAGGGCACACTAGAACCAGAATAT

TTCAATTCAGTGTGTAGACTTATGAAAACTATAGGTCCAGACATGTTCCT

CGGAACTTGTCGGCGTTGTCCTGCTGAAATTGTTGACACTGTGAGTGCTT

TGGTTTATGATAATAAGCTTAAAGCACATAAAGACAAATCAGCTCAATGC

TTTAAAATGTTTTATAAGGGTGTTATCACGCATGATGTTTCATCTGCAAT

TAACAGGCCACAAATAGGCGTGGTAAGAGAATTCCTTACACGTAACCCTG

CTTGGAGAAAAGCTGTCTTTATTTCACCTTATAATTCACAGAATGCTGTA

GCCTCAAAGATTTTGGGACTACCAACTCAAACTGTTGATTCATCACAGGG

CTCAGAATATGACTATGTCATATTCACTCAAACCACTGAAACAGCTCACT

CTTGTAATGTAAACAGATTTAATGTTGCTATTACCAGAGCAAAAGTAGGC

ATACTTTGCATAATGTCTGATAGAGACCTTTATGACAAGTTGCAATTTAC

AAGTCTTGAAATTCCACGTAGGAATGTGGCAACTTTACAAGCTGAAAATG

TAACAGGACTCTTTAAAGATTGTAGTAAGGTAATCACTGGGTTACATCCT

ACACAGGCACCTACACACCTCAGTGTTGACACTAAATTCAAAACTGAAGG

TTTATGTGTTGACATACCTGGCATACCTAAGGACATGACCTATAGAAGAC

TCATCTCTATGATGGGTTTTAAAATGAATTATCAAGTTAATGGTTACCCT

AACATGTTTATCACCCGCGAAGAAGCTATAAGACATGTACGTGCATGGAT

TGGCTTCGATGTCGAGGGGTGTCATGCTACTAGAGAAGCTGTTGGTACCA

ATTTACCTTTACAGCTAGGTTTTTCTACAGGTGTTAACCTAGTTGCTGTA

CCTACAGGTTATGTTGATACACCTAATAATACAGATTTTTCCAGAGTTAG

TGCTAAACCACCGCCTGGAGATCAATTTAAACACCTCATACCACTTATGT

ACAAAGGACTTCCTTGGAATGTAGTGCGTATAAAGATTGTACAAATGTTA

AGTGACACACTTAAAAATCTCTCTGACAGAGTCGTATTTGTCTTATGGGC

ACATGGCTTTGAGTTGACATCTATGAAGTATTTTGTGAAAATAGGACCTG

AGCGCACCTGTTGTCTATGTGATAGACGTGCCACATGCTTTTCCACTGCT

TCAGACACTTATGCCTGTTGGCATCATTCTATTGGATTTGATTACGTCTA

TAATCCGTTTATGATTGATGTTCAACAATGGGGTTTTACAGGTAACCTAC

AAAGCAACCATGATCTGTATTGTCAAGTCCATGGTAATGCACATGTAGCT

AGTTGTGATGCAATCATGACTAGGTGTCTAGCTGTCCACGAGTGCTTTGT

TAAGCGTGTTGACTGGACTATTGAATATCCTATAATTGGTGATGAACTGA

AGATTAATGCGGCTTGTAGAAAGGTTCAACACATGGTTGTTAAAGCTGCA

TTATTAGCAGACAAATTCCCAGTTCTTCACGACATTGGTAACCCTAAAGC

TATTAAGTGTGTACCTCAAGCTGATGTAGAATGGAAGTTCTATGATGCAC

AGCCTTGTAGTGACAAAGCTTATAAAATAGAAGAATTATTCTATTCTTAT

GCCACACATTCTGACAAATTCACAGATGGTGTATGCCTATTTTGGAATTG

CAATGTCGATAGATATCCTGCTAATTCCATTGTTTGTAGATTTGACACTA

GAGTGCTATCTAACCTTAACTTGCCTGGTTGTGATGGTGGCAGTTTGTAT

GTAAATAAACATGCATTCCACACACCAGCTTTTGATAAAAGTGCTTTTGT

TAATTTAAAACAATTACCATTTTTCTATTACTCTGACAGTCCATGTGAGT

CTCATGGAAAACAAGTAGTGTCAGATATAGATTATGTACCACTAAAGTCT

GCTACGTGTATAACACGTTGCAATTTAGGTGGTGCTGTCTGTAGACATCA

TGCTAATGAGTACAGATTGTATCTCGATGCTTATAACATGATGATCTCAG

CTGGCTTTAGCTTGTGGGTTTACAAACAATTTGATACTTATAACCTCTGG

AACACTTTTACAAGACTTCAGAGTTTAGAAAATGTGGCTTTTAATGTTGT

AAATAAGGGACACTTTGATGGACAACAGGGTGAAGTACCAGTTTCTATCA

TTAATAACACTGTTTACACAAAAGTTGATGGTGTTGATGTAGAATTGTTT

GAAAATAAAACAACATTACCTGTTAATGTAGCATTTGAGCTTTGGGCTAA

GCGCAACATTAAACCAGTACCAGAGGTGAAAATACTCAATAATTTGGGTG

TGGACATTGCTGCTAATACTGTGATCTGGGACTACAAAAGAGATGCTCCA

GCACATATATCTACTATTGGTGTTTGTTCTATGACTGACATAGCCAAGAA

ACCAACTGAAACGATTTGTGCACCACTCACTGTCTTTTTTGATGGTAGAG

TTGATGGTCAAGTAGACTTATTTAGAAATGCCCGTAATGGTGTTCTTATT

ACAGAAGGTAGTGTTAAAGGTTTACAACCATCTGTAGGTCCCAAACAAGC

TAGTCTTAATGGAGTCACATTAATTGGAGAAGCCGTAAAAACACAGTTCA

ATTATTATAAGAAAGTTGATGGTGTTGTCCAACAATTACCTGAAACTTAC

TTTACTCAGAGTAGAAATTTACAAGAATTTAAACCCAGGAGTCAAATGGA

AATTGATTTCTTAGAATTAGCTATGGATGAATTCATTGAACGGTATAAAT

TAGAAGGCTATGCCTTCGAACATATCGTTTATGGAGATTTTAGTCATAGT

CAGTTAGGTGGTTTACATCTACTGATTGGACTAGCTAAACGTTTTAAGGA

ATCACCTTTTGAATTAGAAGATTTTATTCCTATGGACAGTACAGTTAAAA

ACTATTTCATAACAGATGCGCAAACAGGTTCATCTAAGTGTGTGTGTTCT

GTTATTGATTTATTACTTGATGATTTTGTTGAAATAATAAAATCCCAAGA

TTTATCTGTAGTTTCTAAGGTTGTCAAAGTGACTATTGACTATACAGAAA

TTTCATTTATGCTTTGGTGTAAAGATGGCCATGTAGAAACATTTTACCCA

AAATTACAATCTAGTCAAGCGTGGCAACCGGGTGTTGCTATGCCTAATCT

TTACAAAATGCAAAGAATGCTATTAGAAAAGTGTGACCTTCAAAATTATG

GTGATAGTGCAACATTACCTAAAGGCATAATGATGAATGTCGCAAAATAT

ACTCAACTGTGTCAATATTTAAACACATTAACATTAGCTGTACCCTATAA

TATGAGAGTTATACATTTTGGTGCTGGTTCTGATAAAGGAGTTGCACCAG

GTACAGCTGTTTTAAGACAGTGGTTGCCTACGGGTACGCTGCTTGTCGAT

TCAGATCTTAATGACTTTGTCTCTGATGCAGATTCAACTTTGATTGGTGA

TTGTGCAACTGTACATACAGCTAATAAATGGGATCTCATTATTAGTGATA

TGTACGACCCTAAGACTAAAAATGTTACAAAAGAAAATGACTCTAAAGAG

GGTTTTTTCACTTACATTTGTGGGTTTATACAACAAAAGCTAGCTCTTGG

AGGTTCCGTGGCTATAAAGATAACAGAACATTCTTGGAATGCTGATCTTT

ATAAGCTCATGGGACACTTCGCATGGTGGACAGCCTTTGTTACTAATGTG

AATGCGTCATCATCTGAAGCATTTTTAATTGGATGTAATTATCTTGGCAA

ACCACGCGAACAAATAGATGGTTATGTCATGCATGCAAATTACATATTTT

GGAGGAATACAAATCCAATTCAGTTGTCTTCCTATTCTTTATTTGACATG

AGTAAATTTCCCCTTAAATTAAGGGGTACTGCTGTTATGTCTTTAAAAGA

AGGTCAAATCAATGATATGATTTTATCTCTTCTTAGTAAAGGTAGACTTA

TAATTAGAGAAAACAACAGAGTTGTTATTTCTAGTGATGTTCTTGTTAAC

AACTAAACGAACAATGTTTGTTTTTCTTGTTTTATTGCCACTAGTCTCTA

GTCAGTGTGTTAATCTTACAACCAGAACTCAATTACCCCCTGCATACACT

AATTCTTTCACACGTGGTGTTTATTACCCTGACAAAGTTTTCAGATCCTC

AGTTTTACATTCAACTCAGGACTTGTTCTTACCTTTCTTTTCCAATGTTA

CTTGGTTCCATGCTATACATGTCTCTGGGACCAATGGTACTAAGAGGTTT

GATAACCCTGTCCTACCATTTAATGATGGTGTTTATTTTGCTTCCACTGA

GAAGTCTAACATAATAAGAGGCTGGATTTTTGGTACTACTTTAGATTCGA

AGACCCAGTCCCTACTTATTGTTAATAACGCTACTAATGTTGTTATTAAA

GTCTGTGAATTTCAATTTTGTAATGATCCATTTTTGGGTGTTTATTACCA

CAAAAACAACAAAAGTTGGATGGAAAGTGAGTTCAGAGTTTATTCTAGTG

CGAATAATTGCACTTTTGAATATGTCTCTCAGCCTTTTCTTATGGACCTT

GAAGGAAAACAGGGTAATTTCAAAAATCTTAGGGAATTTGTGTTTAAGAA

TATTGATGGTTATTTTAAAATATATTCTAAGCACACGCCTATTAATTTAG

TGCGTGATCTCCCTCAGGGTTTTTCGGCTTTAGAACCATTGGTAGATTTG

CCAATAGGTATTAACATCACTAGGTTTCAAACTTTACTTGCTTTACATAG

AAGTTATTTGACTCCTGGTGATTCTTCTTCAGGTTGGACAGCTGGTGCTG

CAGCTTATTATGTGGGTTATCTTCAACCTAGGACTTTTCTATTAAAATAT

AATGAAAATGGAACCATTACAGATGCTGTAGACTGTGCACTTGACCCTCT

CTCAGAAACAAAGTGTACGTTGAAATCCTTCACTGTAGAAAAAGGAATCT

ATCAAACTTCTAACTTTAGAGTCCAACCAACAGAATCTATTGTTAGATTT

CCTAATATTACAAACTTGTGCCCTTTTGGTGAAGTTTTTAACGCCACCAG

ATTTGCATCTGTTTATGCTTGGAACAGGAAGAGAATCAGCAACTGTGTTG

CTGATTATTCTGTCCTATATAATTCCGCATCATTTTCCACTTTTAAGTGT

TATGGAGTGTCTCCTACTAAATTAAATGATCTCTGCTTTACTAATGTCTA

TGCAGATTCATTTGTAATTAGAGGTGATGAAGTCAGACAAATCGCTCCAG

GGCAAACTGGAAAGATTGCTGATTATAATTATAAATTACCAGATGATTTT

ACAGGCTGCGTTATAGCTTGGAATTCTAACAATCTTGATTCTAAGGTTGG

TGGTAATTATAATTACCTGTATAGATTGTTTAGGAAGTCTAATCTCAAAC

CTTTTGAGAGAGATATTTCAACTGAAATCTATCAGGCCGGTAGCACACCT

TGTAATGGTGTTGAAGGTTTTAATTGTTACTTTCCTTTACAATCATATGG

TTTCCAACCCACTAATGGTGTTGGTTACCAACCATACAGAGTAGTAGTAC

TTTCTTTTGAACTTCTACATGCACCAGCAACTGTTTGTGGACCTAAAAAG

TCTACTAATTTGGTTAAAAACAAATGTGTCAATTTCAACTTCAATGGTTT

AACAGGCACAGGTGTTCTTACTGAGTCTAACAAAAAGTTTCTGCCTTTCC

AACAATTTGGCAGAGACATTGCTGACACTACTGATGCTGTCCGTGATCCA

CAAACACTTGAGATTCTTGACATTACACCATGTTCTTTTGGTGGTGTCAA

TGTTATAACACCAGGAACAAATACTTCTAACCAGGTTGCTGTTCTTTATC

ATGATGTTAACTGCACAGAAGTCCCTGTTGCTATTCATGCAGATCAACTT

ACTCCTACTTGGCGTGTTTATTCTACAGGTTCTAATGTTTTTCAAACACG

TGCAGGCTGTTTAATAGGGGCTGAACATGTCAACAACTCATATGAGTGTG

ACATACCCATTGGTGCAGGTATATGCGCTAGTTATCAGACTCAGACTAAT

TCTCCTCGGCGGGCACGTAGTGTAGCTAGTCAATCCATCATTGCCTACAC

TATGTCACTTGGTGCAAAAAATTCAGTTGCTTACTCTAATAACTCTATTG

CCATACCCACAAATTTTACTATTAGTGTTACCACAGAAATTCTACCAGTG

TCTATGACCAAGACATCAGTAGATTGTACAATGTACATTTGTGGTGATTC

AACTGAATGCAGCAATCTTTTGTTGCAATATGGCAGTTTTTGTACACAAT

TAAACCGTGCTTTAACTGGAATAGTGTTGAACAAGACAAAAACACCCAAG

AAGTTTTTGCACAAGTCAAACAAATTTACAAAACACCACCAATTAAAGAT

TTTGGTGGTTTTAATTTTTCACAAATATTACCAGATCCATCAAAACCAAG

CAAGAGGTCATTTATTGAAGATCTACTTTTCAACAAAGTGACACTTGCAG

ATGCTGGCTTCATCAAACAATATGGTGATTGCCTTGGTGATATTGCTGCT

AGAGACCTCATTTGTGCACAAAAGTTTAACGGCCTTACTGTTTTGCCACC

TTTGCTCACAGATGAAATGATTGCTCAATACACTTCTGCACTGTTAGCGG

GTACAATCACTTCTGGTTGGACCTTTGGTGCAGGTGCTGCATTACAAATA

CCATTTGCTATGCAAATGGCTTATAGGTTTAATGGTATTGGAGTTACACA

GAATGTTCTTTATGAGAACCAAAAATTGATTGCCAACCAATTTAATAGTG

CTATTGGCAAAATTCAAGACTCACTTTCTTCCACAGCAAGTGCACTTGGA

AAACTTCAAGATGTGGTCAACCAAAATGCACAAGCTTTAAACACGCTTGT

TAAACAACTTAGCTCCAATTTTGGTGCAATTTCAAGTGTTTTAAATGATA

TCCTTTCACGTCTTGACAAAGTTGAGGCTGAAGTGCAAATTGATAGGTTG

ATCACAGGCAGACTTCAAAGTTTGCAGACATATGTGACTCAACAATTAAT

TAGAGTTGCAGAAATCAGAGCTTCTGCTAATCTTGCTGCTACTAAAATGT

CAGAGTGTGTACTTGGACAATCAAAAAGAGTTGATTTTTGTGGAAAGGGC

TATCATCTTATGTCCTTCCTTCAGTCAGCACCTCATGGTGTAGTATTCTT

GCATGTGATTATGTCCCTGCACAAGAAAAGAACTTCACAACTGCTCCTGC

CATTTGTCATGATGGAAAAGCACACTTTCCTCGTGAAGGTGTCTTTGTTT

CAAATGGCACACACTGGTTTGTAACACAAAGGAATTTTTATGAACCACAA

ATCATTACTACAGACAACACATTTGTGTCTGGTAACTGTGATGTTGTAAT

AGGAATTGTCAACAACACAGTTTATGATCCTTTGCAACCTGAATTAGACT

CATTCAAGGAGGAGTTAGATAAATATTTTAAGAATCATACATCACCAGAT

GTTGATTTAGGTGACATCTCTGGCATTAATGCTTCAGTTGTAAACATTCA

AAAAGAAATTGACCGCCTCAATGAGGTTGCCAAGAATTTAAATGAATCTC

TCATCGATCTCCAAGAACTTGGAAAGTATGAGCAGTATATAAAATGGCCA

TGGTACATTTGGCTAGGTTTTATAGCTGGCTTGATTGCCATAGTAATGGT

GACAATTATGCTTTGCTGTATGACCAGTTGCTGTAGTTGTCTCAAGGGCT

GTTGTTCTTGTGGATCCTGCTGCAAATTTGATGAAGACGACTCTGAGCCA

GTGCTCAAAGGAGTCAAATTACATTACACATAAACGAACTTATGGATTTG

TTTATGAGAATCTTCACAATTGGAACTGTAACTTTGAAGCAAGGTGAAAT

CAAGGATGCTACTCCTTCAGATTTTGTTCGCGCTACTGCAACGATACCGA

TACAAGCCTCACTCCCTTTCGGATGGCTTATTGTTGGCGTTGCACTTCTT

GCTGTTTTTCAGAGCGCTTCCAAAATCATAACCCTCAAAAAGAGATGGCA

ACTAGCACTCTCCAAGGGTGTTCACTTTGTTTGCAACTTGCTGTTGTTGT

TTGTAACAGTTTACTCACACCTTTTGCTCGTTGCTGCTGGCCTTGAAGCC

CCTTTTCTCTATCTTTATGCTTTAGTCTACTTCTTGCAGAGTATAAACTT

TGTAAGAATAATAATGAGGCTTTGGCTTTGCTGGAAATGCCGTTCCAAAA

ACCCATTACTTTATGATGCCAACTATTTTCTTTGCTGGCATACTAATTGT

TACGACTATTGTATACCTTACAATAGTGTAACTTCTTCAATTGTCATTAC

TTCAGGTGATGGCACAACAAGTCCTATTTCTGAACATGACTACCAGATTG

GTGGTTATACTGAAAAATGGGAATCTGGAGTAAAAGACTGTGTTGTATTA

CACAGTTACTTCACTTCAGACTATTACCAGCTGTACTCAACTCAATTGAG

TACAGACACTGGTGTTGAACATGTTACCTTCTTCATCTACAATAAAATTG

TTGATGAGCCTGAAGAACATGTCCAAATTCACACAATCGACGGTTCATCC

GGAGTTGTTAATCCAGTAATGGAACCAATTTATGATGAACCGACGACGAC

TACTAGCGTGCCTTTGTAAGCACAAGCTGATGAGTACGAACTTATGTACT

CATTCGTTTCGGAAGAGACAGGTACGTTAATAGTTAATAGCGTACTTCTT

TTTCTTGCTTTCGTGGTATTCTTGCTAGTTACACTAGCCATCCTTACTGC

GCTTCGATTGTGTGCGTACTGCTGCAATATTGTTAACGTGAGTCTTGTAA

AACCTTCTTTTTACGTTTACTCTCGTGTTAAAAATCTGAATTCTTCTAGA

GTTCCTGATCTTCTGGTCTAAACGAACTAAATATTATATTAGTTTTTCTG

TTTGGAACTTTAATTTTAGCCATGGCAGATTCCAACGGTACTATTACCGT

TGAAGAGCTTAAAAAGCTCCTTGAACAATGGAACCTAGTAATAGGTTTCC

TATTCCTTACATGGATTTGTCTTCTACAATTTGCCTATGCCAACAGGAAT

AGGTTTTTGTATATAATTAAGTTAATTTTCCTCTGGCTGTTATGGCCAGT

AACTTTAGCTTGTTTTGTGCTTGCTGCTGTTTACAGAATAAATTGGATCA

CCGGTGGAATTGCTATCGCAATGGCTTGTCTTGTAGGCTTGATGTGGCTC

AGCTACTTCATTGCTTCTTTCAAACTGTTTGCGCGTACGCGTTCCATGTG

GTCATTCAATCCAAAAACTAACATTCTTCTCAACGTGCCACTCCATGGCA

CTATTCTGACCAGACCGCTTCTAGAAAGTGAACTCGAAATCGGAGCTGTG

ATCCTTCGTGGACATCTTCATATAGCTGGACACCATCTAGGACGCTGTGA

CATCAAGGACCTGCCTAAAGAAATCACTGTTGCTACATCACGAACGCTTT

CTTATTACAAATTGGGAGCTTCGCAGCGTGTAGCAGGTGACTCAGGTTTT

GCTGCATACAGTCGCTACAGGATTGGCAACTATAAATTAAACACAGACCA

TTCCAGTAGCAGTGACAATATTGCTTTGCTTGTACAGTAAGTGACAACAG

ATGTTTCATCTCGTTGACTTTCAGGTTACTATAGCAGAGATATTATATTA

CTAATTATTATGAGGACTTTTAAAGTTTCCATTTGGAATCTTGATTACAT

CATAAACCTCATAATTAAAAATTTATCTAAGTCACTAACTGAGAATAAAT

ATTCTCAATTAGATGAAGAGCAACCAATGGAGATTGATTAAACGAACATG

AAAATTATTCTTTTCTTGGCACTGATAACACTCGCTACTTGTGAGCTTTA

TCACTACCAAGAGTGTGTTAGAGGTACAACAGTACTTTTAAAAGAACCTT

GCTCTTCTGGAACATACGAGGGCAATTCACCATTTCATCCTCTAGCTGAT

AACAAATTTGCACTGACTTGCTTTAGCACTCAATTTGCTTTTGCTTGTCC

TGACGGCGTAAAACACGTCTATCAGTTACGTGCCAGATCAGTTTCACCTA

AACTGTTCATCAGACAAGAGGAAGTTCAAGAACTTTACTCTCCAATTTTT

CTTATTGTTGCGGCAATAGTGTTTATAACACTTTGCTTCACACTCAAAAG

AAAGACAGAATGATTGAACTTTCATTAATTGACTTCTATTTGTGCTTTTT

AGCCTTTCTGCTATTCCTTGTTTTAATTATGCTTATTATCTTTTGGTTCT

CACTTGAACTGCAAGATCATAATGAAACTTGTCACGCCTAAACGAACATG

AAATTTCTTGTTTTCTTAGGAATCATCACAACTGTAGCTGCATTTCACCA

AGAATGTAGTTTACAGTCATGTACTCAACATCAACCATATGTAGTTGATG

ACCCGTGTCCTATTCACTTCTATTCTAAATGGTATATTAGAGTAGGAGCT

AGAAAATCAGCACCTTTAATTGAATTGTGCGTGGATGAGGCTGGTTCTAA

ATCACCCATTCAGTACATCGATATCGGTAATTATACAGTTTCCTGTTTAC

CTTTTACAATTAATTGCCAGGAACCTAAATTGGGTAGTCTTGTAGTGCGT

TGTTCGTTCTATGAAGACTTTTTAGAGTATCATGACGTTCGTGTTGTTTT

AGATTTCATCTAAACGAACAAACTAAAATGTCTGATAATGGACCCCAAAA

TCAGCGAAATGCACCCCGCATTACGTTTGGTGGACCCTCAGATTCAACTG

GCAGTAACCAGAATGGAGAACGCAGTGGGGCGCGATCAAAACAACGTCGG

CCCCAAGGTTTACCCAATAATACTGCGTCTTGGTTCACCGCTCTCACTCA

ACATGGCAAGGAAGACCTTAAATTCCCTCGAGGACAAGGCGTTCCAATTA

ACACCAATAGCAGTCCAGATGACCAAATTGGCTACTACCGAAGAGCTACC

AGACGAATTCGTGGTGGTGACGGTAAAATGAAAGATCTCAGTCCAAGATG

GTATTTCTACTACCTAGGAACTGGGCCAGAAGCTGGACTTCCCTATGGTG

CTAACAAAGACGGCATCATATGGGTTGCAACTGAGGGAGCCTTGAATACA

CCAAAAGATCACATTGGCACCCGCAATCCTGCTAACAATGCTGCAATCGT

GCTACAACTTCCTCAAGGAACAACATTGCCAAAAGGCTTCTACGCAGAAG

GGAGCAGAGGCGGCAGTCAAGCCTCTTCTCGTTCCTCATCACGTAGTCGC

AACAGTTCAAGAAATTCAACTCCAGGCAGCAGTAGGGGAACTTCTCCTGC

TAGAATGGCTGGCAATGGCGGTGATGCTGCTCTTGCTTTGCTGCTGCTTG

ACAGATTGAACCAGCTTGAGAGCAAAATGTCTGGTAAAGGCCAACAACAA

CAAGGCCAAACTGTCACTAAGAAATCTGCTGCTGAGGCTTCTAAGAAGCC

TCGGCAAAAACGTACTGCCACTAAAGCATACAATGTAACACAAGCTTTCG

GCAGACGTGGTCCAGAACAAACCCAAGGAAATTTTGGGGACCAGGAACTA

ATCAGACAAGGAACTGATTACAAACATTGGCCGCAAATTGCACAATTTGC

CCCCAGCGCTTCAGCGTTCTTCGGAATGTCGCGCATTGGCATGGAAGTCA

CACCTTCGGGAACGTGGTTGACCTACACAGGTGCCATCAAATTGGATGAC

AAAGATCCAAATTTCAAAGATCAAGTCATTTTGCTGAATAAGCATATTGA

CGCATACAAAACATTCCCACCAACAGAGCCTAAAAAGGACAAAAAGAAGA

AGGCTGATGAAACTCAAGCCTTACCGCAGAGACAGAAGAAACAGCAAACT

GTGACTCTTCTTCCTGCTGCAGATTTGGATGATTTCTCCAAACAATTGCA

ACAATCCATGAGCAGTGCTGACTCAACTCAGGCCTAAACTCATGCAGACC

ACACAAGGCAGATGGGCTATATAAACGTTTTCGCTTTTCCGTTTACGATA

TATAGTCTACTCTTGTGCAGAATGAATTCTCGTAACTACATAGCACAAGT

AGATGTAGTTAACTTTAATCTCACATAGCAATCTTTAATCAGTGTGTAAC

ATTAGGGAGGACTTGAAAGAGCCACCACATTTTCACCGAGGCCACGCGGA

GTACGATCGAGTGTACAGTGAACAATGCTAGGGAGAGCTGCCTATATGGA

AGAGCCCTAATGTGTAAAATTAATTTTAGTAGTGCTATCCCCATGTGATT

TTAATAGCTTCTTAGGAGAATGACAAAAAAA
